# A network meta-analysis for efficacies and toxicities of different concurrent chemoradiotherapy regimens in the treatment of locally advanced non-small cell lung cancer

**DOI:** 10.1186/s12885-022-09717-8

**Published:** 2022-06-20

**Authors:** Qiangqiang Zheng, Shihui Min, Yunfeng Zhou

**Affiliations:** 1 Department of Thoracic Surgery, West China School of Public Health and West China Fourth Hospital, Sichuan University, Chengdu, Sichuan 610044 People’s Republic of China; 2 Department of Oncology, West China School of Public Health and West China Fourth Hospital, Sichuan University, Chengdu, Sichuan 610044 People’s Republic of China

**Keywords:** Locally advanced non-small cell lung cancer, Concurrent chemoradiotherapy, Efficacy, Toxicity, Randomized controlled trial, Network meta-analysis

## Abstract

**Background:**

Concurrent chemoradiotherapy (CCRT) has become the cornerstone of treatment for patients with locally advanced non-small cell lung cancer (LA-NSCLC). The aim of this study was to compare the efficacies and toxicities of different CCRT regimens in the treatment of LA-NSCLC by adopting a network meta-analysis (NMA).

**Methods:**

An exhaustive search of PubMed, EMBASE, Web of Science, and the Cochrane Central Register of Controlled Trials (CENTRAL) was conducted to identify relevant studies from inception to October 1, 2020. Direct and indirect evidence was combined to calculate the odds radios (ORs) and 95% confidence intervals (CIs), as well as to plot the surface under the cumulative ranking (SUCRA) curves. Cluster analyses were adopted to compare the efficacies and toxicities of different CCRT regimens according to the similarity of 2 variables. Publication bias was detected by comparison-adjusted funnel plots.

**Results:**

Twenty-two studies were enrolled in this NMA, including 18 regimens: CCRT (cisplatin + etoposide), CCRT (carboplatin + paclitaxel), CCRT (pemetrexed + carboplatin), CCRT (pemetrexed + cisplatin), CCRT (docetaxel + cisplatin), CCRT (S-1 + cisplatin), CCRT (mitomycin + vindesine + cisplatin), CCRT (cisplatin + vinorelbine), CCRT (cisplatin), CCRT (etoposide + cisplatin + amifostine), RT, CCRT (5-FU), CCRT (paclitaxel + cisplatin), CCRT (irinotecan + carboplatin), CCRT (nedaplatin), CCRT (carboplatin + etoposide), CCRT (paclitaxel), and CCRT (carboplatin). The results indicated that the regimens with CCRT (cisplatin + etoposide), CCRT (carboplatin + paclitaxel), CCRT (pemetrexed + cisplatin), CCRT (S-1 + cisplatin), and CCRT (cisplatin + vinorelbine) had relatively better efficacies compared with other regimens. As for toxicities of different CCRT regimens, the CCRT (carboplatin + paclitaxel), CCRT (pemetrexed + cisplatin), and CCRT (docetaxel + cisplatin) were relatively lower.

**Conclusions:**

Our study demonstrated that CCRT (pemetrexed + cisplatin) and CCRT (carboplatin + paclitaxel) might be the best options for the treatment of LA-NSCLC, and CCRT (pemetrexed + cisplatin) had the highest 3-year overall survival (OS) rate.

## Introduction

Lung cancer is the most common malignancy worldwide and the leading cause of cancer-related deaths [1, 2]. In many countries, lung cancer remains a major public health threat with a high overall incidence and high frequency of diagnosis [1, 2]. Every year, 2.2 million people are diagnosed with lung cancer and 1.6 million die from the disease around the world [3]. In China, lung cancer has the highest number of new cases and deaths of all cancers (820, 000 and 710, 000, respectively), accounting for 17.9 and 23.8% of all cancer incidence and mortality [3]. No-small cell lung cancer (NSCLC) approximately accounts for 85% of all lung cancer [2, 3], and approximately 30% of patients are diagnosed with locally advanced NSCLC (LA-NSCLC) [4]. Currently, greater effort in encouraging smoking cessation, screening in high-risk individuals, and prompt diagnostic procedures have significantly brought a rise of early-stage disease [5], but the most common presentation among non-metastatic patients remains LA-NSCLC.

Over the past decade, molecular oncology has rapidly elucidated so-called “driver mutations” in NSCLC, leading to the emergence of targeted therapies. While the initial clinical trials did not demonstrate a survival benefit with EGFR-TKIs for the overall population of advanced NSCLC patients [6], a specific subset of patients carrying mutations on the kinase domain of EGFR gene were found to be highly sensitive to the targeted drugs [7]. However, targeted therapy is ineffective in patients with negative driver genes. Therefore, for the unresectable patients with negative driver genes, combined-modality therapy using chemotherapy and thoracic radiation therapy is very crucial [8, 9]. The Radiation Therapy Oncology Group (RTOG) trial 0617 compared standard-dose (SD, 60 Gy) versus high-dose (HD, 74 Gy) radiation with concurrent chemotherapy for stage III NSCLC. Median overall survival (OS) was 28.7 and 20.3 months in the SD and HD arms, respectively, 5-year OS and progression-free survival (PFS) rates were 32.1% and 23% and 18.3% and 13%, respectively [10]. Consequently, the concurrent chemoradiotherapy (CCRT) regimen has been recommended as current standard therapeutic paradigms of care for patients with unresectable LA-NSCLC [11]. Meanwhile, radiation therapy has become more effective and safe, the RTOG 94–10 trial proved that CCRT arm with once-daily RT had a better median OS (17.0 months) [12]. Although the CCRT is considered the standard care [8] and a variety of CCRT combinations and schedules are currently used, and current guidelines list many regimens as recommended therapeutic options for LA-NSCLC. However, the adverse effects of certain agents, which lead to failure to complete the scheduled regimen, extension of chemotherapy intervals or reduction of the recommended dosage, have limited their clinical application, and the optimal chemotherapy regimen remains unclear. Therefore, it is extremely important to investigate and identify effective CCRT regimen with low toxicity.

Network meta-analysis (NMA), also known as multiple-treatments comparison (MTC), enables us to synthesize data from direct (within-trail) comparisons and can provide indirect (inter-trail) comparisons of multiple treatment regimens when direct comparisons are unavailable [13]. In addition, the cluster analysis enables us to estimate the rank probability that, witch of the treatments is the best, the second best, etc. Thus, we performed a NMA to compare the efficacies and toxicities of different CCRT regimens and to research which is the best regimen in the treatment of LA-NSCLC.

## Materials and methods

### Literature and database search strategy

We performed systematic literature search of PubMed, EMBASE (via Ovid interface), Web of Science (via campus network of Sichuan University), and the Cochrane Central Register of Controlled Trials (CENTRAL) (via Ovid interface) from their incipiency to October 1, 2020. We used the Medical Subject Headings/Emtree combined with free text words of locally advanced non-small lung cancer, LA-NSCLC, concurrent chemoradiotherapy, concurrent chemotherapy and radiotherapy, cisplatin, docetaxel, pemetrexed, carboplatin, S-1, etoposide, amifostine, vinorelbine, paclitaxel, nedaplatin, mitomycin, vindesine, irinotecan, 5-FU, and RT. Additionally, reference lists of eligible published clinical trials and meta-analyses were also tracked manually to identify other relevant studies. Only studies published in English were included. All the initially identified articles were scrutinized independently by two reviewers (Qiangqiang Zheng and Shihui Min).

### Selection criteria

Eligible studies were selected according to the following inclusion criteria: (a) the study design was a randomized controlled trials (RCT), (b) different chemotherapeutic interventions were included, (c) research subjects were patients with LA-NSCLC, (d) literature containing the following outcome measures, including overall response rate (ORR), 1-year OS rate, 2-year OS rate, 3-year OS rate, and toxicities (anemia, leukopenia, neutropenia, thrombocytopenia, febrile neutropenia, pneumonitis, nausea, vomiting, and esophagitis). Studies were excluded if the following criteria were met: (a) the articles were meta-analyses, letters, reviews, editorial materials, meeting abstracts, case reports, and expert opinions, (b) not human studies, (c) not RCT, (d) not English literature, (e) patients who received surgery or adjuvant chemotherapy, (f) studies without adequate information about efficacy and toxicity. Two authors (Qiangqiang Zheng and Shihui Min) independently assessed the titles and abstracts of studies to identify whether these studies met the inclusion criteria. In the case of existing discrepancies, the two authors reached consensus via discussion.

### Data extraction and quality assessment

Data were extracted from the selected studies by two independent investigators (Qiangqiang Zheng and Shihui Min). The following information were extracted: (a) publication data including first author, publication year, country, sample size, and therapeutic regimens, (b) the efficacy of different CCRT regimens in the treatment of LA-NSCLC, including ORR, 1-year OS rate, 2-year OS rate, and 3-year OS rate, (c) the toxicity of different CCRT regimens, including anemia, leukopenia, neutropenia, thrombocytopenia, febrile neutropenia, pneumonitis, nausea, vomiting, and esophagitis. Newcastle–Ottawa Quality Assessment Scale (NOS) was used to estimate the quality of every original study [14]. Three perspectives including selection, comparability, and ascertainment of exposure and outcomes were considered for a semi-quantitative estimation. A study with NOS ≥ 6 was regarded as a high-quality study [15].

### Data synthesis and analysis

To assess the efficacy and toxicity of different CCRT regimens in the treatment of LA-NSCLC, we determined odds radios (ORs) and their 95% confidence intervals (CIs) under a fixed effect model or a random effect model as the appropriate summarized statistics, and the Z-test was performed to detect the significance of the pooled effect size [16].

Heterogeneity among the pooled studies was evaluated by Cochran Q-statistic and I^2^ test [17]. Random effect model was used when significant heterogeneity existed among studies (*P* < 0.1 or I^2^ > 50%). Otherwise, a fixed effect model was employed.

A network evidence plot was drawn with the nodes indicating interventions, the node size representing sample size, and the thickness of lines referring to the accuracy of the effect size of the comparison between 2 studies.

A surface under the cumulative ranking (SUCRA) curve was used to compare the SUCRA value of different CCRT regimens to ascertain the efficacy and toxicity ranks, the larger the SUCRA value, the better the efficacy or the lower the toxicity [18]. Cluster analyses were adopted to compare efficacy and toxicity of different CCRT regimens according to the similarity of 2 variables [18].

A comparison-adjusted funnel plot was used to evaluate the small-study effect, which considered the difference of the summary effect for each set of studies [19].

Finally, we declared that all of the above statistical analyses were accomplished by STATA (version 14.0) (Stata Corporation, College Station, TX, USA). All statistical tests were two-tailed with a *P* < 0.05 being considered statistically significant.

## Results

### Overview of the literature search

**Fig. 1:**
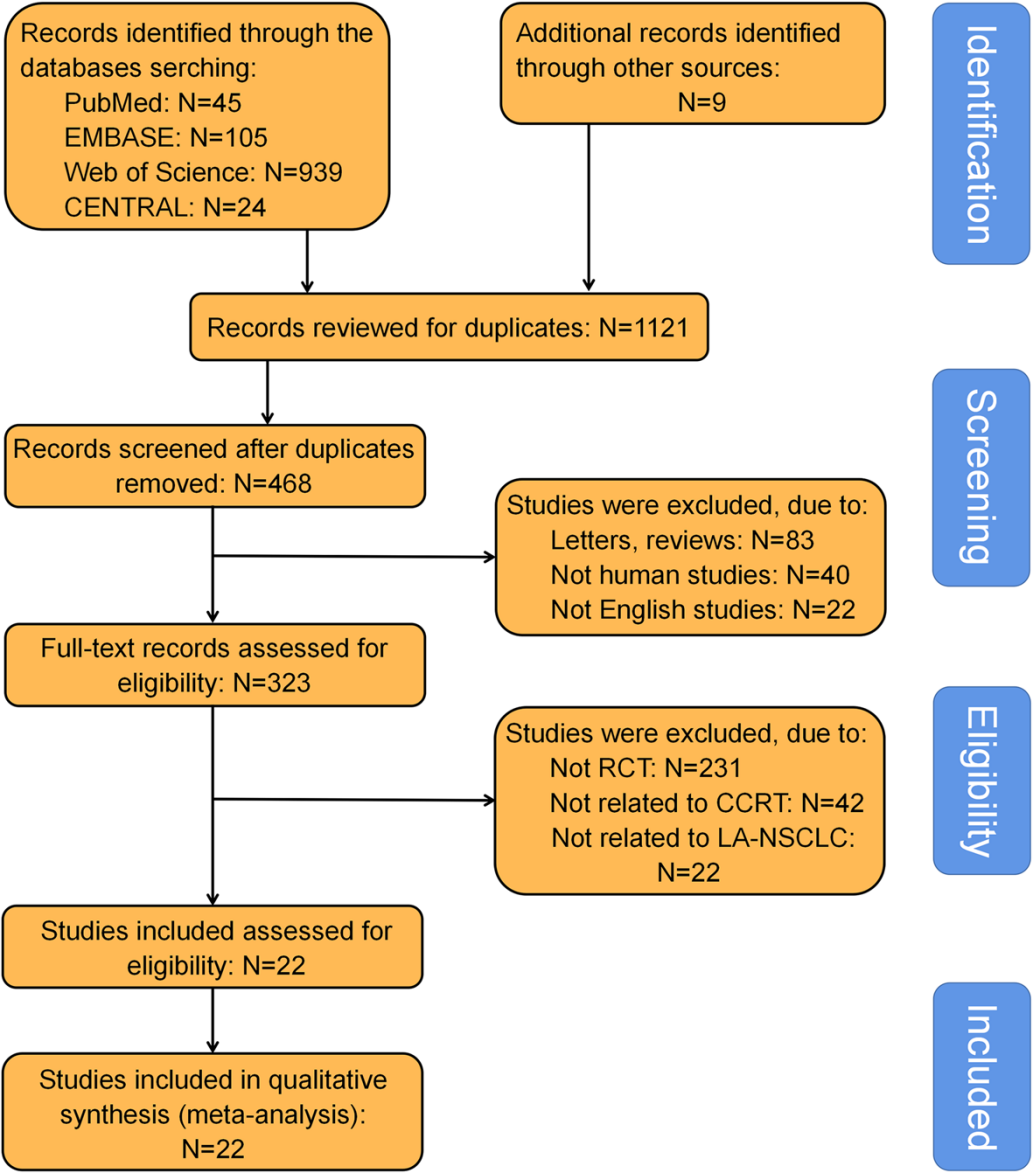
Flowchart of the study selection process. *CCRT* = *concurrent chemoradiotherapy. CENTRAL* = *Cochrane central register of controlled trials. RCT* = *randomized controlled trial. LA-NSCLC* = *local advanced non-small cell lung cancer*

The study selection process was shown in Fig. 1. Computer-based database searches and complementary manual search retrieved a total of 1,032 relevant articles. After removing 564 duplicates, we read the titles and abstracts of the 468 studies left, 145 studies were excluded because they either did not English studies (*n* = 22), or did not human studies (*n* = 40), or were letters, reviews, meta-analyses, case reports, and meeting abstracts (*n* = 83). After meticulously reading, 301 studies were excluded because 231 studies were not RCT, 42 studies were not related to CCRT, and 28 studies were not related to LA-NSCLC. In total, 22 eligible studies [20–41] with 3,178 patients were enrolled in this NMA.

### Characteristics of the included studies

Twenty-two articles published between 1992 and 2017, consisting of a total number of 3,178 participants with LA-NSCLC were included in this NMA. There were 21 two-arm studies and 1 three-arm study with 20 comparisons. Briefly, study sample sizes ranged from 22 to 555. From the 22 studies, 10 studies were conducted in Caucasians, and the other 12 studies were Asians. Among all individuals, 507 were treated with CCRT (cisplatin + etoposide) regimen, 227 with CCRT (carboplatin + paclitaxel) regimen, 348 with CCRT (pemetrexed + cisplatin) regimen, 254 with CCRT (mitomycin + vindesine + cisplatin) regimen, 229 with CCRT (cisplatin) regimen, 584 with RT, and remaining 1029 were treated with other CCRT regimens. The detailed characteristics of the included studies were diaplayed in Table 1.

**Table 1:** Main characteristics of included studies in this NMA

First Author	Year	Country	Ethnicity	Interventions			Sample Size				Age, y		
				T1	T2	T3	Total	T1	T2	T3	T1	T2	T3
Chen F [20]	2017	China	Asians	K	O	—	107	47	60	—	67 (60–80)	68 (60–80)	—
Liang J [21]	2017	China	Asians	A	M	—	191	95	96	—	NR	NR	—
Feng JF [22]	2016	China	Asians	F	I	—	72	36	36	—	63 (42–84)	62 (42–83)	—
Sen F [23]	2016	Turkey	Caucasians	A	E	—	105	50	55	—	54 (32–70)	55 (37–73)	—
Senan S [24]	2016	USA	Caucasians	D	A	—	555	283	272	—	58.7 (34.6–80.4)	59.5 (28.0–83.7)	—
Zhao Q [25]	2016	China	Asians	H	D	—	100	48	52	—	57.4 (34–73)	60.3 (40–75)	—
Yao L [26]	2015	China	Asians	F	I	—	40	20	20	—	59.6 (40.3–78.9)	60.4 (40.3–80.5)	—
Liew MS [27]	2013	Australia	Caucasians	B	A	—	75	44	31	—	71 (44–83)	63 (32–76)	—
Sugawara S [28]	2013	Japan	Asians	F	H	—	66	35	31	—	NR	NR	—
Atagi S [29]	2012	Japan	Asians	K	R	—	197	98	99	—	77 (71–93)	77 (71–89)	—
Wang LH [30]	2012	China	Asians	A	B	—	65	33	32	—	55.4 (26–77)	60.9 (40–75)	—
John H [31]	2010	USA	Caucasians	C	D	—	22	9	13	—	62 (46–74)	60 (43–84)	—
Segawa Y [32]	2010	Japan	Asians	E	G	—	200	99	101	—	NR	NR	—
Yamamoto N [33]	2010	Japan	Asians	G	N	B	456	153	152	151	63.3 (31–74)	62 (30–74)	63.0 (38–74)
Atagi S [34]	2005	Japan	Asians	K	R	—	46	23	23	—	77 (72–84)	77 (71–83)	—
Cakir S [35]	2004	Turkey	Caucasians	K	I	—	176	88	88	—	61 (47–70)	60 (46–70)	—
Sarihan S [36]	2004	Turkey	Caucasians	K	Q	—	41	20	21	—	63 (37–77)	55 (36–68)	—
Komaki R [37]	2002	USA	Caucasians	J	A	—	53	27	26	—	62 (37–77)	64 (43–74)	—
Ball D [38]	1999	Australia	Caucasians	K	R	—	107	53	54	—	65 (40–78)	66 (46–77)	—
Ball D [39]	1997	Australia	Caucasians	K	L	—	200	101	99	—	67 (40–91)	67 (42–86)	—
Jeremic B [40]	1996	Japan	Asians	K	P	—	131	66	65	—	58 (46–65)	59 (42–67)	—
Trovo MG [41]	1992	Italy	Caucasians	K	I	—	173	88	85	—	61 (43–70)	62 (36–69)	—

A, CCRT (cisplatin + etoposide); B, CCRT (carboplatin + paclitaxel); C, CCRT (pemetrexed + carboplatin); CCRT, concurrent chemoradiotherapy; D, CCRT (pemetrexed + cisplatin); E, CCRT (docetaxel + cisplatin); F, CCRT (S-1 + cisplatin); G, CCRT (mitomycin + vindesine + cisplatin); H, CCRT (cisplatin + vinorelbine); I, CCRT (cisplatin); J, CCRT (etoposide + cisplatin + amifostine); K, RT; L, CCRT (5-FU); M, CCRT (paclitaxel + cisplatin); N, CCRT (irinotecan + carboplatin); *NMA* Network meta-analysis, *NR* Not report; O, CCRT (nedaplatin); P, CCRT (carboplatin + etoposide); Q, CCRT (paclitaxel); R, CCRT (carboplatin)

### Quality assessment

Two researchers were assigned to evaluate all of the included studies. The results of the quality assessment involving 22 RCTs were presented in Table 2. The mean NOS score was 8.4 (range from 7 to 9), which suggested a good quality level.

**Table 2:** Newcastle–Ottawa Quality Assessment Scale (NOS) scoring records of the included studies

First Author[Year]	Selection	Comparability	Exposure			Total score
			Assessment of outcome	Follow-up long enough for outcome	Adequacy of follow-up of cohorts	
Chen F [20]	4	2	1	1	1	9
Liang J [21]	4	2	1	1	1	9
Feng JF [22]	4	2	1	1	1	9
Sen F [23]	4	2	1	1	1	9
Senan S [24]	4	2	1	1	0	8
Zhao Q [25]	4	2	1	1	1	9
Yao L [26]	4	2	1	1	0	8
Liew MS [27]	4	2	1	1	1	9
Sugawara S [28]	4	2	1	1	1	9
Atagi S [29]	4	2	1	1	1	9
Wang LH [30]	4	2	1	1	0	8
John H [31]	4	2	1	0	0	7
Segawa Y [32]	4	2	1	1	1	9
Yamamoto N [33]	4	2	1	1	1	9
Atagi S [34]	4	2	1	0	0	7
Cakir S [35]	4	2	1	1	1	9
Sarihan S [36]	4	2	1	0	0	7
Komaki R [37]	4	2	1	0	0	7
Ball D [38]	4	2	1	1	1	9
Ball D [39]	4	2	1	1	1	9
Jeremic B [40]	4	2	1	1	1	9
Trovo MG [41]	4	2	1	0	0	7

### Pairwise meta-analyses for efficacies of different CCRT regimens

Random effect models were used to perform direct pairwise matchings, and the results of efficacies were shown in Table 3. The results demonstrated that RT had a worse efficacy in ORR compared with CCRT (5-FU) and CCRT (nedaplatin) (OR = 0.45, 95% CI = 0.23–0.90; OR = 0.38, 95% CI = 0.17–0.85; respectively). For the 1-year OS rate, CCRT (docetaxel + cisplatin) had a worse efficacy compared with CCRT (mitomycin + vindesine + cisplatin) (OR = 0.45, 95% CI = 0.23–0.87), CCRT (cisplatin) had a better efficacy compared with the RT (OR = 4.08, 95% CI = 2.16–7.72), and RT had a worse efficacy compared with CCRT (nedaplatin) (OR = 0.39, 95% CI = 0.17–0.90). As for 2-year OS rate, CCRT (S-1 + cisplatin) had a better efficacy compared with CCRT (cisplatin) (OR = 3.15, 95% CI = 1.43–6.96), CCRT (etoposide + cisplatin) had a better efficacy compared with CCRT (docetaxel + cisplatin) (OR = 3.32, 95% CI = 1.47–7.51), CCRT (paclitaxel + carboplatin) had a better efficacy compared with CCRT (irinotecan + carboplatin) (OR = 1.32, 95% CI = 0.84–2.09), CCRT (cisplatin) had a better efficacy compared with the RT (OR = 5.78, 95% CI = 1.88–17.80), and RT had a worse efficacy compared with CCRT (carboplatin + etoposide) (OR = 0.46, 95% CI = 0.22–0.96). In terms of 3-year OS rate, CCRT (S-1 + cisplatin) had a better efficacy compared with CCRT (cisplatin) (OR = 3.08, 95% CI = 1.37–6.94), RT had a worse efficacy compared with CCRT (carboplatin) (OR = 0.42, 95% CI = 0.21–0.83), CCRT (etoposide + cisplatin) had a better efficacy compared with CCRT (docetaxel + cisplatin) and CCRT (paclitaxel + cisplatin) (OR = 3.09, 95% CI = 1.38–6.88; OR = 1.98, 95% CI = 1.07–3.65; respectively), CCRT (paclitaxel + carboplatin) had a better efficacy compared with CCRT (irinotecan + carboplatin) (OR = 1.70, 95% CI = 1.03–2.79), and CCRT (cisplatin) had a better efficacy compared with RT (OR = 4.90, 95% CI = 1.03–23.37).

**Table 3:** Pairwise meta-analyses for efficacies of different CCRT regimens in the treatment of LA-NSCLC

Included studies	Comparisons	Efficacy events		Pairwise meta-analysis		
		Treatment 1	Treatment 2	OR (95% CI)	I^2^	*P*
**ORR**						
3 studies	K VS. R	84/174	95/176	0.80 (0.52–1.21)	0.0%	0.756
2 studies	F VS. I	34/56	29/56	1.44 (0.68–3.05)	0.0%	0.935
2 studies	I VS. K	99/173	92/176	1.22 (0.80–1.86)	84.0%	0.012
1 study	A VS. B	21/33	26/32	0.40 (0.13–1.26)	NA	NA
1 study	A VS. D	90/272	102/283	0.88 (0.62–1.25)	NA	NA
1 study	A VS. E	40/50	44/55	1.00 (0.38–2.61)	NA	NA
1 study	A VS. M	70/95	62/96	1.54 (0.83–2.85)	NA	NA
1 study	B VS. G	96/151	102/153	0.87 (0.54–1.40)	NA	NA
1 study	B VS. N	96/151	86/152	1.34 (0.84–2.12)	NA	NA
1 study	C VS. D	1/9	6/13	0.15 (0.01–1.53)	NA	NA
1 study	D VS. H	46/52	44/48	0.70 (0.18–2.64)	NA	NA
1 study	E VS. G	78/99	71/101	1.57 (0.82–2.99)	NA	NA
1 study	G VS. N	102/153	86/152	1.53 (0.96–2.44)	NA	NA
1 study	F VS. H	28/35	22/31	1.64 (0.53–5.09)	NA	NA
1 study	K VS. L	16/101	29/99	**0.45 (0.23–0.90)**	NA	NA
1 study	K VS. O	24/47	44/60	**0.38 (0.17–0.85)**	NA	NA
1 study	K VS. P	56/66	60/65	0.47 (0.15–1.45)	NA	NA
1 study	K VS. Q	14/20	18/21	0.39 (0.08–1.84)	NA	NA
**1-year OS rate**						
3 studies	K VS. R	110/174	119/176	0.82 (0.53–1.28)	0.0%	0.962
2 studies	A VS. B	39/64	49/76	0.86 (0.43–1.71)	72.4%	0.057
2 studies	F VS. I	50/56	47/56	1.60 (0.53–4.83)	0.0%	0.995
1 study	A VS. D	209/272	215/283	1.05 (0.71–1.55)	NA	NA
1 study	A VS. E	45/50	45/55	2.00 (0.63–6.32)	NA	NA
1 study	A VS. M	71/95	77/96	0.73 (0.37–1.44)	NA	NA
1 study	B VS. G	121/151	116/153	1.29 (0.75–2.22)	NA	NA
1 study	B VS. N	121/151	114/152	1.34 (0.78–2.31)	NA	NA
1 study	D VS. H	44/52	37/48	1.64 (0.60–4.49)	NA	NA
1 study	E VS. G	17/99	32/101	**0.45 (0.23–0.87)**	NA	NA
1 study	G VS. N	116/153	114/152	1.05 (0.62–1.76)	NA	NA
1 study	F VS. H	27/35	23/31	1.17 (0.38–3.62)	NA	NA
1 study	I VS. K	52/88	23/88	**4.08 (2.16–7.72)**	NA	NA
1 study	K VS. L	26/101	26/99	0.97 (0.52–1.83)	NA	NA
1 study	K VS. O	12/47	28/60	**0.39 (0.17–0.90)**	NA	NA
1 study	K VS. P	45/66	48/65	0.76 (0.36–1.62)	NA	NA
**2-year OS rate**						
2 studies	A VS. B	23/64	24/76	1.22 (0.60–2.46)	71.4%	0.061
2 studies	K VS. R	48/151	62/153	0.68 (0.43–1.10)	16.7%	0.273
2 studies	F VS. I	41/56	26/56	**3.15 (1.43–6.96)**	0.0%	0.854
1 study	A VS. D	141/272	147/283	1.00 (0.71–1.39)	NA	NA
1 study	A VS. E	36/50	24/55	**3.32 (1.47–7.51)**	NA	NA
1 study	A VS. M	46/95	41/96	1.26 (0.71–2.23)	NA	NA
1 study	B VS. G	71/151	73/153	0.97 (0.62–1.53)	NA	NA
1 study	B VS. N	71/151	61/152	1.32 (0.84–2.09)	NA	NA
1 study	D VS. H	28/52	27/48	0.91 (0.41–2.00)	NA	NA
1 study	E VS. G	39/99	52/101	0.61 (0.35–1.07)	NA	NA
1 study	F VS. H	18/35	15/31	1.13 (0.43–2.97)	NA	NA
1 study	G VS. N	73/153	61/152	1.36 (0.86–2.14)	NA	NA
1 study	I VS. K	19/88	4/88	**5.78 (1.88–17.80)**	NA	NA
1 study	K VS. L	4/101	9/99	0.42 (0.12–1.40)	NA	NA
1 study	K VS. O	6/47	15/60	0.43 (0.15–1.21)	NA	NA
1 study	K VS. P	17/66	28/65	**0.46 (0.22–0.96)**	NA	NA
**3-year OS rate**						
2 studies	F VS. I	27/56	13/56	**3.08 (1.37–6.94)**	0.0%	0.921
2 studies	K VS. R	14/151	30/153	**0.42 (0.21–0.83)**	0.0%	0.412
1 study	A VS. B	11/33	4/32	3.50 (0.98–12.50)	NA	NA
1 study	A VS. D	101/272	113/283	0.89 (0.63–1.25)	NA	NA
1 study	A VS. E	29/50	17/55	**3.09 (1.38–6.88)**	NA	NA
1 study	A VS. M	39/95	25/96	**1.98 (1.07–3.65)**	NA	NA
1 study	B VS. G	40/151	54/153	0.66 (0.40–1.08)	NA	NA
1 study	B VS. N	40/151	37/152	1.12 (0.67–1.88)	NA	NA
1 study	D VS. H	23/52	15/48	1.74 (0.77–3.96)	NA	NA
1 study	E VS. G	62/99	61/101	1.10 (0.62–1.94)	NA	NA
1 study	F VS. H	12/35	10/31	1.10 (0.39–3.06)	NA	NA
1 study	G VS. N	54/153	37/152	**1.70 (1.03–2.79)**	NA	NA
1 study	I VS. K	9/88	2/88	**4.90 (1.03–23.37)**	NA	NA
1 study	K VS. L	1/101	2/99	0.49 (0.04–5.44)	NA	NA
1 study	K VS. O	4/47	9/60	0.53 (0.15–1.83)	NA	NA
1 study	K VS. P	7/66	15/65	0.40 (0.15–1.05)	NA	NA

A, CCRT (cisplatin + etoposide); B, CCRT (carboplatin + paclitaxel); C, CCRT (pemetrexed + carboplatin); *CCRT* Concurrent chemoradiotherapy, *CI* Confidence interval; D, CCRT (pemetrexed + cisplatin); E, CCRT (docetaxel + cisplatin); F, CCRT (S-1 + cisplatin); G, CCRT (mitomycin + vindesine + cisplatin); H, CCRT (cisplatin + vinorelbine); I, CCRT (cisplatin); K, RT; L, CCRT (5-FU); *LA-NSCLC* Locally advanced non-small cell lung cancer; M, CCRT (paclitaxel + cisplatin); N, CCRT (irinotecan + carboplatin); *NA* Not available; O, CCRT (nedaplatin); *OR* Odds radios, *ORR* Overall response rate; P, CCRT (carboplatin + etoposide); Q, CCRT (paclitaxel); R, CCRT (carboplatin)

### Pairwise meta-analyses for hematological toxicities of different CCRT regimens

We conducted direct-paired comparisons of incidences of hematological toxicities, and the results were displayed in Table 4. Compared with CCRT (carboplatin), the incidence of leukopenia with RT was relatively lower (OR = 0.01, 95%CI = 0.00–0.05). Compared with CCRT (mitomycin + vindesine + cisplatin), the incidences of anemia, neutropenia, thrombocytopenia, and febrile neutropenia with CCRT (carboplatin + paclitaxel) were relatively lower (OR = 0.34, 95% CI = 0.15–0.76; OR = 0.02, 95% CI = 0.01–0.04; OR = 0.20, 95% CI = 0.09–0.44; OR = 0.08, 95% CI = 0.03–0.20; respectively). Compared with CCRT (carboplatin + paclitaxel), the incidence of neutropenia with CCRT (etoposide + cisplatin) was relatively higher (OR = 3.16, 95% CI = 1.57–6.34). Compared with CCRT (pemetrexed + cisplatin), the incidences of neutropenia and febrile neutropenia with CCRT (etoposide + cisplatin) were relatively higher (OR = 1.79, 95% CI = 1.20–2.66; OR = 2.42, 95% CI = 1.08–5.40; respectively). Compared with CCRT (irinotecan + carboplatin), the incidences of neutropenia and thrombocytopenia with CCRT (carboplatin + paclitaxel) were relatively lower (OR = 0.26, 95% CI = 0.16–0.42; OR = 0.42, 95% CI = 0.18–0.99; respectively). Compared with CCRT (mitomycin + vindesine + cisplatin), the incidences of neutropenia, thrombocytopenia, and febrile neutropenia with CCRT (docetaxel + cisplatin) were relatively lower (OR = 0.10, 95% CI = 0.04–0.26; OR = 0.06, 95% CI = 0.01–0.27; OR = 0.45, 95% CI = 0.24–0.84; respectively). Compared with CCRT (pemetrexed + cisplatin) and CCRT (S-1 + cisplatin), the incidences of leukopenia with CCRT (vinorelbine + cisplatin) were relatively lower (OR = 0.38, 95% CI = 0.16–0.90; OR = 0.19, 95% CI = 0.06–0.55; respectively). Compared with CCRT (irinotecan + carboplatin), the incidences of neutropenia and thrombocytopenia with CCRT (mitomycin + vindesine + cisplatin) were relatively higher (OR = 13.66, 95% CI = 6.48–28.77; OR = 2.13, 95% CI = 1.14–3.96; respectively). Compared with CCRT (pemetrexed + cisplatin), the incidences of febrile neutropenia with CCRT (mitomycin + vindesine + cisplatin) were relatively higher (OR = 7.05, 95% CI = 3.31–15.01).

**Table 4:** Pairwise meta-analyses for hematological toxicities of different CCRT regimens in the treatment of LA-NSCLC

Included studies	Comparisons	Toxicity events		Pairwise meta-analysis		
		Treatment 1	Treatment 2	OR (95% CI)	I^2^	*P*
**Anemia**						
2 studies	A VS. B	5/64	5/76	1.20 (0.33–4.36)	0.0%	0.807
2 studies	F VS. I	5/56	5/56	1.00 (0.27–3.67)	0.0%	0.508
3 studies	K VS. R	3/174	9/176	0.33 (0.09–1.22)	0.0%	0.649
1 study	A VS. D	22/272	16/283	1.47 (0.75–2.86)	NA	NA
1 study	A VS. E	1/50	1/55	1.10 (0.07–18.10)	NA	NA
1 study	A VS. M	1/95	1/96	1.01 (0.06–16.40)	NA	NA
1 study	B VS. G	9/151	24/153	**0.34 (0.15–0.76)**	NA	NA
1 study	B VS. N	9/151	13/152	0.68 (0.28–1.65)	NA	NA
1 study	D VS. H	5/52	8/48	0.53 (0.16–1.76)	NA	NA
1 study	F VS. H	2/35	2/31	0.88 (0.12–6.64)	NA	NA
1 study	G VS. N	24/153	13/152	1.99 (0.97–4.07)	NA	NA
1 study	I VS. K	8/88	7/88	1.16 (0.40–3.34)	NA	NA
**Leukopenia**						
2 studies	F VS. I	13/56	10/56	1.39 (0.55–3.50)	0.0%	0.943
2 studies	K VS. R	2/121	74/122	**0.01 (0.00–0.05)**	73.6%	0.052
1 study	A VS. D	69/272	53/283	1.48 (0.98–2.21)	NA	NA
1 study	A VS. M	29/95	26/96	1.18 (0.63–2.21)	NA	NA
1 study	D VS. H	11/52	20/48	**0.38 (0.16–0.90)**	NA	NA
1 study	F VS. H	8/35	19/31	**0.19 (0.06–0.55)**	NA	NA
1 study	I VS. K	48/88	46/88	1.10 (0.61–1.98)	NA	NA
1 study	K VS. O	1/47	21/60	**0.04 (0.01–0.31)**	NA	NA
**Neutropenia**						
2 studies	A VS. B	37/64	23/76	**3.16 (1.57–6.34)**	0.0%	0.636
1 study	A VS. D	78/272	52/283	**1.79 (1.20–2.66)**	NA	NA
1 study	A VS. E	4/50	1/55	4.70 (0.51–43.51)	NA	NA
1 study	B VS. G	35/151	144/153	**0.02 (0.01–0.04)**	NA	NA
1 study	B VS. N	35/151	82/152	**0.26 (0.16–0.42)**	NA	NA
1 study	E VS. G	61/99	94/101	**0.10 (0.04–0.26)**	NA	NA
1 study	G VS. N	144/153	82/152	**13.66 (6.48–28.77)**	NA	NA
**Thrombocytopenia**						
2 studies	A VS. B	8/64	5/76	2.03 (0.63–6.54)	0.0%	0.345
2 studies	F VS. I	10/56	9/56	1.14 (0.42–3.05)	0.0%	0.834
1 study	A VS. D	19/272	15/283	1.34 (0.67–2.70)	NA	NA
1 study	A VS. E	1/50	1/55	1.10 (0.07–18.10)	NA	NA
1 study	A VS. M	1/95	1/96	1.01 (0.06–16.40)	NA	NA
1 study	B VS. G	8/151	34/153	**0.20 (0.09–0.44)**	NA	NA
1 study	B VS. N	8/151	18/152	**0.42 (0.18–0.99)**	NA	NA
1 study	D VS. H	3/52	3/48	0.92 (0.18–4.79)	NA	NA
1 study	E VS. G	2/99	25/101	**0.06 (0.01–0.27)**	NA	NA
1 study	G VS. N	34/153	18/152	**2.13 (1.14–3.96)**	NA	NA
1 study	F VS. H	1/35	1/31	0.88 (0.05–14.73)	NA	NA
**Febrile neutropenia**						
1 study	A VS. B	6/31	5/44	1.87 (0.52–6.79)	NA	NA
1 study	A VS. D	20/272	9/283	**2.42 (1.08–5.40)**	NA	NA
1 study	B VS. G	5/151	47/153	**0.08 (0.03–0.20)**	NA	NA
1 study	B VS. N	5/151	9/152	0.54 (0.18–1.66)	NA	NA
1 study	E VS. G	22/99	39/101	**0.45 (0.24–0.84)**	NA	NA
1 study	G VS. N	47/153	9/152	**7.05 (3.31–15.01)**	NA	NA

A, CCRT (cisplatin + etoposide); B, CCRT (carboplatin + paclitaxel); *CCRT* Concurrent chemoradiotherapy; *CI* Confidence interval; D, CCRT (pemetrexed + cisplatin); E, CCRT (docetaxel + cisplatin); F, CCRT (S-1 + cisplatin); G, CCRT (mitomycin + vindesine + cisplatin); H, CCRT (cisplatin + vinorelbine); I, CCRT (cisplatin); K, RT; L, CCRT (5-FU); *LA-NSCLC* Locally advanced non-small cell lung cancer; M, CCRT (paclitaxel + cisplatin); N, CCRT (irinotecan + carboplatin); *NA* Not available; O, CCRT (nedaplatin); *OR* Odds radio; R, CCRT (carboplatin)

### Pairwise meta-analyses for non-hematological toxicities of different CCRT regimens

Pairwise comparisons of non-hematological toxicites were accomplished for the different CCRT regimens, and the results were demonstrated in Table 5. Compared with CCRT (mitomycin + vindesine + cisplatin), the incidences of nausea/vomiting and pneumonitis with CCRT (carboplatin + paclitaxel) were relatively lower (OR = 0.09, 95% CI = 0.03–0.23; OR = 0.40, 95% CI = 0.21–0.76; respectively). Compared with CCRT (irinotecan + carboplatin) regimen, the incidences of nausea/vomiting and pneumonitis with CCRT (mitomycin + vindesine + cisplatin) were relatively higher (OR = 8.10, 95% CI = 3.51–18.69; OR = 2.08, 95% CI = 1.13–3.83; respectively). Compared with CCRT (paclitaxel + cisplatin), the incidence of esophagitis with CCRT (etoposide + cisplatin) was relatively higher (OR = 3.75, 95% CI = 1.43–9.87). Compared with CCRT (irinotecan + carboplatin), the incidence of esophagitis with CCRT (carboplatin + paclitaxel) was relatively higher (OR = 3.90, 95% CI = 1.07–14.28). Compared with CCRT (5-FU), the incidence of esophagitis with RT was relatively lower (OR = 0.24, 95% CI = 0.07–0.91). Compared with CCRT (paclitaxel + cisplatin), the incidence of pneumonitis with CCRT (etoposide + cisplatin) was relatively lower (OR = 0.47, 95% CI = 0.24–0.91).

**Table 5:** Pairwise meta-analyses for non-hematological toxicities of different CCRT regimens in the treatment of LA-NSCLC

Included studies	Comparisons	Toxicity events		Pairwise meta-analysis		
		Treatment 1	Treatment 2	OR (95% CI)	I^2^	*P*
**Nausea/vomiting**						
2 studies	E VS. I	6/56	5/56	1.22 (0.35–4.27)	0.0%	0.810
2 studies	I VS. K	26/173	21/176	1.32 (0.70–2.47)	0.0%	0.887
2 studies	J VS. R	1/121	1/122	1.01 (0.06–16.31)	0.0%	0.996
1 study	A VS. B	2/31	1/44	2.97 (0.26–34.24)	NA	NA
1 study	A VS. D	24/272	20/283	1.27 (0.69–2.36)	NA	NA
1 study	A VS. E	1/50	2/55	0.54 (0.05–6.15)	NA	NA
1 study	A VS. M	11/95	19/96	0.53 (0.24–1.19)	NA	NA
1 study	B VS. G	5/151	43/153	**0.09 (0.03–0.23)**	NA	NA
1 study	B VS. N	5/151	7/152	0.71 (0.22–2.29)	NA	NA
1 study	D VS. H	3/52	1/48	2.88 (0.29–28.65)	NA	NA
1 study	E VS. G	8/99	5/101	1.69 (0.53–5.35)	NA	NA
1 study	G VS. N	43/153	7/152	**8.10 (3.51–18.69)**	NA	NA
1 study	F VS. H	4/35	2/31	1.87 (0.32–11.00)	NA	NA
1 study	K VS. L	3/101	7/99	0.40 (0.10–1.60)	NA	NA
1 study	K VS. O	2/47	19/60	0.22 (0.02–2.03)	NA	NA
**Esophagitis**						
2 studies	A VS. B	23/64	28/76	0.96 (0.48–1.92)	0.0%	0.733
3 studies	K VS. R	8/174	14/176	0.56 (0.23–1.37)	0.0%	0.902
2 studies	F VS. I	4/56	2/56	2.08 (0.36–11.83)	0.0%	0.989
2 studies	I VS. K	22/173	14/176	1.69 (0.83–3.41)	0.0%	0.439
1 study	A VS. D	21/272	22/283	0.99 (0.53–1.85)	NA	NA
1 study	A VS. E	3/50	3/55	1.11 (0.21–5.75)	NA	NA
1 study	A VS. M	19/95	6/96	**3.75 (1.43–9.87)**	NA	NA
1 study	B VS. G	11/151	6/153	1.92 (0.69–5.35)	NA	NA
1 study	B VS. N	11/151	3/152	**3.90 (1.07–14.28)**	NA	NA
1 study	C VS. D	1/9	1/13	1.50 (0.08–27.61)	NA	NA
1 study	D VS. H	1/52	3/48	0.29 (0.03–2.93)	NA	NA
1 study	E VS. G	14/99	6/101	2.61 (0.96–7.09)	NA	NA
1 study	F VS. H	1/35	1/31	0.88 (0.05–14.73)	NA	NA
1 study	G VS. N	6/153	3/152	2.03 (0.50–8.26)	NA	NA
1 study	K VS. L	3/101	11/99	**0.24 (0.07–0.91)**	NA	NA
1 study	K VS. O	2/47	5/60	0.49 (0.09–2.64)	NA	NA
1 study	K VS. P	7/66	8/65	0.85 (029–2.48)	NA	NA
1 study	K VS. Q	1/20	1/21	1.05 (0.06–18.05)	NA	NA
**Pneumonitis**						
2 studies	A VS. B	10/64	18/76	0.60 (0.25–1.40)	40.9%	0.193
2 studies	F VS. I	3/56	2/56	1.53 (0.25–9.52)	0.0%	0.706
2 studies	K VS. R	8/121	14/122	0.55 (0.22–1.35)	0.0%	0.648
1 study	A VS. D	7/272	6/283	1.22 (0.40–3.68)	NA	NA
1 study	A VS. E	1/50	6/55	0.17 (0.02–1.35)	NA	NA
1 study	A VS. J	6/26	1/27	7.80 (0.87–70.10)	NA	NA
1 study	A VS. M	18/95	32/96	**0.47 (0.24–0.91)**	NA	NA
1 study	B VS. G	16/151	35/153	**0.40 (0.21–0.76)**	NA	NA
1 study	B VS. N	16/151	19/152	0.83 (0.41–1.68)	NA	NA
1 study	C VS. D	1/9	1/13	1.50 (0.08–27.61)	NA	NA
1 study	D VS. H	1/52	1/48	0.92 (0.06–15.15)	NA	NA
1 study	E VS. G	10/99	7/101	1.51 (0.55–4.14)	NA	NA
1 study	F VS. H	2/35	4/31	0.41 (0.07–2.41)	NA	NA
1 study	G VS. N	35/153	19/152	**2.08 (1.13–3.83)**	NA	NA
1 study	I VS. K	24/88	22/88	1.13 (0.57–2.21)	NA	NA
1 study	K VS. O	1/47	3/60	0.41 (0.04–4.10)	NA	NA
1 study	K VS. P	5/66	7/65	0.68 (0.19–5.99)	NA	NA
1 study	K VS. Q	3/20	3/21	1.39 (0.62–3.11)	NA	NA

A, CCRT (cisplatin + etoposide); B, CCRT (carboplatin + paclitaxel); C, CCRT (pemetrexed + carboplatin); *CCRT* Concurrent chemoradiotherapy, *CI* Confidence interval; D, CCRT (pemetrexed + cisplatin); E, CCRT (docetaxel + cisplatin); F, CCRT (S-1 + cisplatin); G, CCRT (mitomycin + vindesine + cisplatin); H, CCRT (cisplatin + vinorelbine); I, CCRT (cisplatin); J, CCRT (etoposide + cisplatin + amifostine); K, RT; L, CCRT (5-FU); *LA-NSCLC* Locally advanced non-small cell lung cancer; M, CCRT (paclitaxel + cisplatin); N, CCRT (irinotecan + carboplatin); *NA* Not available; O, CCRT (nedaplatin); *OR* Odds radio; P, CCRT (carboplatin + etoposide); Q, CCRT (paclitaxel); R, CCRT (carboplatin)

### Inconsistency tests for efficacies and toxicities of different CCRT regimens

Inconsistency tests showed that the results of direct and indirect evidences of different CCRT regimens were consistency, the consistency model was adopted (*P* > 0.05) (Tables 6, 7 and 8).

**Table 6:** Inconsistency tests for efficacies of different CCRT regimens in the treatment of LA-NSCLC

Pairwise comparisons	Direct OR values				Indirect OR values				*P* values			
	ORR	1-yera OS rate	2-year OS rate	3-year OS rate	ORR	1-yera OS rate	2-year OS rate	3-year OS rate	ORR	1-yera OS rate	2-year OS rate	3-year OS rate
A VS. B	0.91	0.13	-0.24	-1.25	-0.59	0.36	-0.74	-1.64	0.122	0.772	0.449	0.651
A VS. D	0.13	-0.05	0.01	0.12	-0.15	1.59	1.75	2.10	0.990	0.949	0.948	0.945
A VS. E	1.48	-0.69	-1.20	-1.13	1.49	-0.92	-0.70	-0.74	0.122	0.772	0.449	0.651
B VS. G	0.14	-0.25	0.03	0.41	-0.26	-0.21	-0.08	0.35	0.581	0.916	0.775	0.879
B VS. N	-0.29	-0.30	-0.28	-0.11	-0.53	-0.27	-0.34	-0.15	0.764	0.954	0.882	0.941
C VS. D	1.93	NR	NR	NR	-0.23	NR	NR	NR	0.981	NR	NR	NR
D VS. H	0.36	-0.49	0.10	-0.56	-0.19	1.23	1.94	1.57	0.982	0.948	0.946	0.943
E VS. G	-0.45	0.81	0.49	-0.09	1.04	0.57	0.99	0.29	0.122	0.772	0.449	0.651
F VS. H	-0.49	-0.16	-0.12	-0.09	0.05	-2.00	-2.13	-2.41	0.983	0.947	0.944	0.941
F VS. I	-0.37	-0.47	-1.16	-1.12	-0.87	1.60	1.22	1.61	0.985	0.944	0.939	0.936
G VS. N	-0.43	-0.04	-0.31	-0.53	-0.19	-0.07	-0.25	-0.50	0.764	0.954	0.882	0.941
I VS. K	-0.20	-1.41	-1.75	-1.59	-0.65	0.83	0.91	1.47	0.988	0.943	0.935	0.934
K VS. L	0.79	0.03	0.89	0.72	-0.05	4.53	6.26	6.85	0.997	0.982	0.979	0.976
K VS. O	0.15	1.25	0.91	NR	-0.68	5.75	6.28	NR	0.997	0.982	0.979	NR
K VS. P	0.97	0.94	0.82	0.64	0.13	5.44	6.19	6.76	0.997	0.982	0.979	0.976
K VS. Q	0.76	NR	NR	NR	-0.08	NR	NR	NR	0.997	NR	NR	NR
K VS. R	0.94	0.28	0.78	0.93	0.11	4.78	6.15	7.05	0.997	0.982	0.978	0.976

A, CCRT (cisplatin + etoposide); B, CCRT (carboplatin + paclitaxel); C, CCRT (pemetrexed + carboplatin); CCRT, concurrent chemoradiotherapy; D, CCRT (pemetrexed + cisplatin); E, CCRT (docetaxel + cisplatin); F, CCRT (S-1 + cisplatin); G, CCRT (mitomycin + vindesine + cisplatin); H, CCRT (cisplatin + vinorelbine); I, CCRT (cisplatin); K, RT; L, CCRT (5-FU); *LA-NSCLC* Locally advanced non-small cell lung cancer; N, CCRT (irinotecan + carboplatin); *NR* Not report; O, CCRT (nedaplatin); *OR* Odds radios; *ORR* Objective response rate, *OS* Overall survival; Q, CCRT (paclitaxel); R, CCRT (carboplatin)

**Table 7:** Incosistency tests for hematological toxicities of different CCRT regimens in the treatment of LA-NSCLC

Pairwise comparisons	Direct OR values					Indirect OR values					*P* values				
	Ane	Leu	Neu	Thr	Feb	Ane	Leu	Neu	Thr	Feb	Ane	Leu	Neu	Thr	Feb
A VS. B	-0.29	NR	-1.19	-0.61	-0.63	-0.35	NR	-4.21	-0.07	-1.08	0.999	NR	0.065	0.770	0.993
A VS. D	-0.38	-0.39	NR	-0.29	NR	-0.38	-0.25	NR	0.03	NR	1.000	0.996	NR	0.995	NR
A VS. E	NR	NR	-2.37	-1.21	NR	0.85	NR	NR	-1.75	NR	NR	NR	0.065	0.770	NR
B VS. G	1.08	NR	3.97	1.63	2.56	1.08	NR	3.72	1.67	2.56	1.000	NR	0.675	0.947	1.000
B VS. N	0.39	NR	1.35	0.87	0.61	0.39	NR	1.16	0.90	0.61	1.000	NR	0.752	0.969	1.000
D VS. H	0.63	0.98	NR	0.09	NR	0.61	1.14	NR	0.44	NR	0.999	0.995	NR	0.995	NR
E VS. G	NR	NR	2.12	2.77	0.79	NR	NR	5.34	2.23	3.07	NR	NR	0.045	0.770	0.991
F VS. H	0.13	1.68	NR	0.13	NR	0.18	1.50	NR	0.54	NR	0.999	0.995	NR	0.996	NR
F VS. I	-0.01	-0.33	NR	NR	NR	-0.03	-0.35	NR	NR	NR	0.999	1.000	NR	NR	NR
G VS. N	-0.69	NR	-2.61	-0.75	-1.95	-0.69	NR	-2.42	-0.78	-1.95	1.000	NR	0.752	0.969	1.000
I VS. K	-0.15	-0.09	NR	NR	NR	-0.14	-0.70	NR	NR	NR	1.000	0.987	NR	NR	NR
K VS. O	NR	3.95	NR	NR	NR	NR	0.60	NR	NR	NR	NR	0.983	NR	NR	NR
K VS. R	1.26	3.43	NR	NR	NR	1.31	1.98	NR	NR	NR	1.000	0.988	NR	NR	NR

A, CCRT (cisplatin + etoposide); *Ane* Anemia; B, CCRT (carboplatin + paclitaxel); *CCRT* Concurrent chemoradiotherapy; D, CCRT (pemetrexed + cisplatin); E, CCRT (docetaxel + cisplatin); F, CCRT (S-1 + cisplatin); Feb, febrile neutropenia; G, CCRT (mitomycin + vindesine + cisplatin); H, CCRT (cisplatin + vinorelbine); I, CCRT (cisplatin); K, RT; *LA-NSCLC* Locally advanced non-small cell lung cancer, *Leu* Leukopenia; N, CCRT (irinotecan + carboplatin); *Neu* Neutropenia, *NR* Not report; O, CCRT (nedaplatin); *OR* Odds radios; R, CCRT (carboplatin); *Thr* thrombocytopenia

**Table 8:** Incosistency tests for non-hematological toxicities of different CCRT regimens in the treatment of LA-NSCLC

Pairwise comparisons	Direct OR values			Indirect OR values			*P* values		
	Nausea/vomiting	Esophagitis	Pneumonitis	Nausea/vomiting	Esophagitis	Pneumonitis	Nausea/vomiting	Esophagitis	Pneumonitis
A VS. B	-0.52	-0.18	-0.05	-1.93	-0.40	1.25	0.430	0.839	0.432
A VS. D	-0.24	0.01	-0.20	0.65	-0.91	0.46	0.977	0.970	0.980
A VS. E	1.02	-0.10	2.58	2.44	0.12	1.27	0.430	0.839	0.431
B VS. G	2.43	-0.65	0.92	2.22	-0.76	1.11	0.800	0.900	0.817
B VS. N	0.34	-1.36	0.19	0.24	-1.41	0.29	0.906	0.964	0.905
C VS. D	NR	-0.35	-1.56	NR	0.08	0.07	NR	0.996	0.985
C VS. F	NR	-0.27	-0.11	NR	-0.99	-2.84	NR	0.997	0.989
D VS. H	-1.06	2.09	1.20	-0.11	1.04	2.22	0.976	0.969	0.971
E VS. G	-0.52	-0.96	-0.41	0.90	-0.73	-1.72	0.430	0.839	0.432
F VS. H	-0.63	0.12	0.89	-1.62	1.17	-0.22	0.976	0.970	0.970
F VS. I	-0.20	-0.73	-1.42	0.89	-1.69	-0.09	0.976	0.974	0.967
G VS. N	-2.09	-0.71	-0.73	-1.99	-0.66	-0.84	0.906	0.964	0.905
I VS. K	-0.28	-0.52	-0.12	0.93	-1.33	1.35	0.977	0.980	0.966
K VS. L	0.91	1.41	NR	3.22	-0.03	NR	0.991	0.994	NR
K VS. O	NR	NR	1.10	NR	NR	3.95	NR	NR	0.989
K VS. Q	2.34	0.72	0.88	4.65	-0.72	3.73	0.991	0.994	0.989
K VS. R	NR	0.17	0.39	NR	-1.27	3.24	NR	0.994	0.989

A, CCRT (cisplatin + etoposide); B, CCRT (carboplatin + paclitaxel); C, CCRT (pemetrexed + carboplatin); *CCRT* Concurrent chemoradiotherapy; D, CCRT (pemetrexed + cisplatin); E, CCRT (docetaxel + cisplatin); F, CCRT (S-1 + cisplatin); G, CCRT (mitomycin + vindesine + cisplatin); H, CCRT (cisplatin + vinorelbine); I, CCRT (cisplatin); K, RT; L, CCRT (5-FU); *LA-NSCLC* Locally advanced non-small cell lung cancer; N, CCRT (irinotecan + carboplatin); *NR* Not report; O, CCRT (nedaplatin); *OR* Odds radios; Q, CCRT (paclitaxel); R, CCRT (carboplatin)

Network meta-analyses for efficacies of different CCRT regimens.

**Fig. 2:**
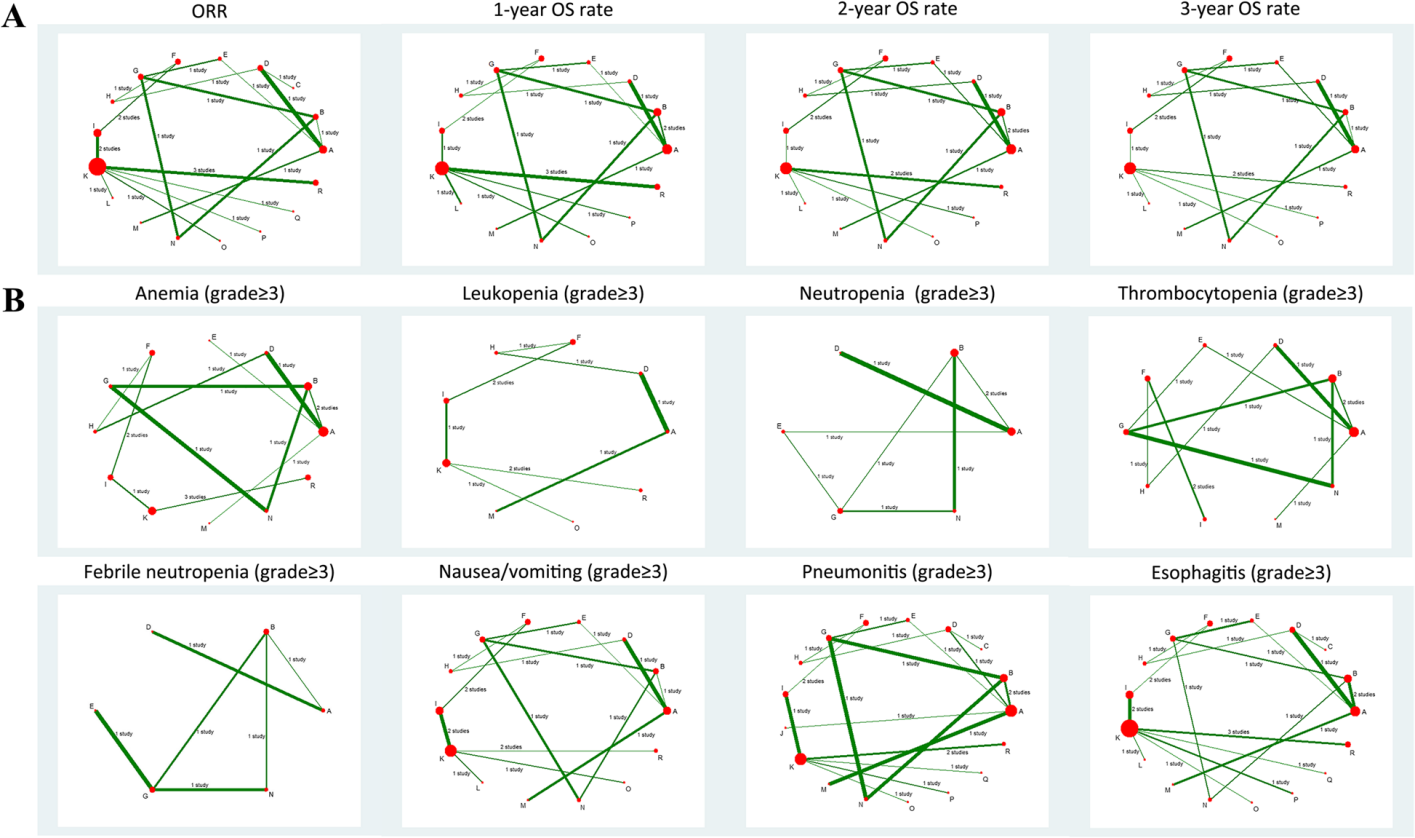
Network evidence plots of different CCRT regimens. **A** Network evidence plots of ORR and OS of different CCRT regimens. **B** Network evidence plots of toxicities of different CCRT regimens. *The width of the lines is proportional to the number of trials comparing every pair of treatments, and the size of every circle is proportional to the number of randomly assigned participants (sample size). A* = *CCRT (cisplatin* + *etoposide). B* = *CCRT (carboplatin* + *paclitaxel). C* = *CCRT (pemetrexed* + *carboplatin). CCRT* = *concurrent chemoradiotherapy. D* = *CCRT (pemetrexed* + *cisplatin). E* = *CCRT (docetaxel* + *cisplatin). F* = *CCRT (S-1* + *cisplatin). G* = *CCRT (mitomycin* + *vindesine* + *cisplatin). H* = *CCRT (cisplatin* + *vinorelbine). I* = *CCRT (cisplatin). J* = *CCRT (etoposide* + *cisplatin* + *amifostine). K* = *RT. L* = *CCRT (5-FU). M* = *CCRT (paclitaxel* + *cisplatin). N* = *CCRT (irinotecan* + *carboplatin). NMA* = *network meta-analysis. O* = *CCRT (nedaplatin). ORR* = *overall response rate. OS* = *overall survival. P* = *CCRT (carboplatin* + *etoposide). Q* = *CCRT (paclitaxel). R* = *CCRT (carboplatin)*

The results of network meta-analyses in efficacies and the network evidence plots were displayed in Table 9 and Fig. 2A. The results suggested that in terms of efficacies, CCRT (cisplatin + etoposide) had better efficacies in 1-year OS rate, 2-year OS rate, and 3-year OS rate than CCRT (5-FU) (OR = 9.49, 95%CI = 1.26–71.34; OR = 14.68, 95% CI = 1.94–110.81; OR = 21.37, 95% CI = 2.31–197.35; respectively). CCRT (cisplatin + pemtrexed) had better efficacies in 1-year OS rate, 2-year OS rate, and 3-year OS rate than RT (OR = 9.04, 95% CI = 1.25–65.44; OR = 14.74, 95% CI = 2.07–105.14; OR = 24.05, 95% CI = 2.67–216.31; respectively). CCRT (S-1 + cisplatin) had better efficacies in 1-year OS rate, 2-year OS rate, and 3-year OS rate than RT (OR = 6.51, 95% CI = 1.81–23.34; OR = 18.37, 95% CI = 4.35–77.48; OR = 15.11, 95% CI = 2.60–87.95; respectively). CCRT (cisplatin + vinorelbine) had better efficacies in 1-year OS rate, 2-year OS rate, and 3-year OS rate than RT (OR = 5.54, 95% CI = 1.01–30.39; OR = 16.25, 95% CI = 2.78–95.14; OR = 13.79, 95% CI = 1.80–105.85; respectively). CCRT (cisplatin) had better efficacies in 1-year OS rate, 2-year OS rate, and 3-year OS rate than RT (OR = 4.08, 95% CI = 2.16–7.72; OR = 5.78, 95% CI = 1.78–18.71; OR = 4.89, 95% CI = 1.03–23.34; respectively). CCRT (carboplatin + paclitaxel) had better efficacies in 1-year OS rate and 2-year OS rate than RT (OR = 10.83, 95% CI = 1.28–91.52; OR = 11.38, 95% CI = 1.29–100.34; respectively). CCRT (S-1 + cisplatin) had better efficacies in 1-year OS rate and 2-year OS rate than CCRT (5-FU) (OR = 6.33, 95% CI = 1.52–26.34; OR = 7.57, 95% CI = 1.12–51.28; respectively). CCRT (S-1 + cisplatin) had better efficacies in 1-year OS rate and 2-year OS rate than CCRT (carboplatin + etoposide) (OR = 4.94, 95% CI = 1.12–21.81; OR = 8.42, 95% CI = 1.61–44.00; respectively). CCRT (S-1 + cisplatin) had better efficacies in 1-year OS rate and 2-year OS rate than CCRT (carboplatin) (OR = 5.35, 95% CI = 1.38–20.68; OR = 12.62, 95% CI = 2.69–59.26; respectively). CCRT (cisplatin) had better efficacies in 1-year OS rate and 2-year OS rate than CCRT (carboplatin) (OR = 3.36, 95% CI = 1.54–7.29; OR = 3.97, 95% CI = 1.08–14.58; respectively). CCRT (carboplatin + paclitaxel) had better efficacies in 2-year OS rate and 3-year OS rate than CCRT (docetaxel + cisplatin) (OR = 3.32, 95% CI = 1.37–8.04; OR = 3.09, 95% CI = 1.38–6.88; respectively). CCRT (pemetrexed + cisplatin) had better efficacies in 2-year OS rate and 3-year OS rate than CCRT (docetaxel + cisplatin) (OR = 3.34, 95% CI = 1.22–9.11; OR = 3.47, 95% CI = 1.45–8.30; respectively). CCRT (pemetrexed + cisplatin) had better efficacies in 2-year OS rate and 3-year OS rate than CCRT (carboplatin) (OR = 10.13, 95% CI = 1.31–78.23; OR = 10.10, 95% CI = 1.01–101.01; respectively). CCRT (S-1 + cisplatin) had better efficacies in 2-year OS rate and 3-year OS rate than CCRT (cisplatin) (OR = 3.18, 95% CI = 1.38–7.31; OR = 3.09, 95% CI = 1.37–6.97; respectively). CCRT (paclitaxel + cisplatin) had better efficacies in 2-year OS rate and 3-year OS rate than RT (OR = 11.65, 95% CI = 1.39–97.88; OR = 10.80, 95% CI = 1.08–108.39; respectively). CCRT (etoposide + cisplatin) had a better efficacy in 1-year OS rate than CCRT (paclitaxel + cisplatin) (OR = 9.24, 95% CI = 1.12–76.48). CCRT (carboplatin + paclitaxel) had better efficacy in 1-year OS rate than CCRT (5-FU) and CCRT (carboplatin) (OR = 10.54, 95% CI = 1.14–97.62; OR = 8.91, 95% CI = 1.01–78.75; respectively). CCRT (pemetrexed + cisplatin) and CCRT (cisplatin) had better efficacies in 1-year OS rate than CCRT (5-FU) (OR = 8.80, 95% CI = 1.10–70.28; OR = 3.97, 95% CI = 1.62–9.75; respectively). CCRT (cisplatin) and CCRT (paclitaxel + cisplatin) had better efficacies in 1-year OS rate than CCRT (carboplatin + etoposide) (OR = 3.10, 95% CI = 1.15–8.34; OR = 9.87, 95% CI = 1.03–94.58; respectively). CCRT (paclitaxel + cisplatin) had a better efficacy in 1-year OS rate than CCRT (carboplatin) (OR = 10.69, 95% CI = 1.21–94.11). CCRT (S-1 + cisplatin) had a better efficacy in 2-year OS rate than CCRT (nedaplatin) (OR = 8.06, 95% CI = 1.32–49.10). CCRT (mitomycin + vindesine + cisplatin) had a better efficacy in 2-year OS rate than RT (OR = 11.70, 95% CI = 1.23–110.88). CCRT (cisplatin + vinorelbine) had better efficacies in 2-year OS rate than CCRT (carboplatin + etoposide) and CCRT (carboplatin) (OR = 7.45, 95% CI = 1.06–52.13; OR = 11.17, 95% CI = 1.75–71.40; respectively). CCRT (cisplatin + etoposide) had a better efficacy in 3-year OS rate than CCRT (irinotecan + carboplatin) (OR = 1.98, 95% CI = 1.07–3.65). CCRT (pemetrexed + cisplatin) had a better efficacy in 3-year OS rate than CCRT (carboplatin + paclitaxel) (OR = 3.94, 95% CI = 1.05–14.72). CCRT (carboplatin + paclitaxel) had better efficacies in 3-year OS rate than CCRT (cisplatin), CCRT (paclitaxel + cisplatin), CCRT (irinotecan + carboplatin), and CCRT (nedaplatin) (OR = 4.91, 95% CI = 1.05–23.03; OR = 2.23, 95% CI = 1.10–4.49; OR = 4.41, 95% CI = 1.07–18.18; OR = 12.68, 95% CI = 1.01–158.37; respectively). CCRT (carboplatin) had a better efficacy in 3-year OS rate than RT (OR = 2.38, 95% CI = 1.19–4.75). However, the ORRs of all CCRT regimens had no differences.

**Table 9:** Network meta-analyses for efficacies of different CCRT regimens in the treatment of LA-NSCLC

OR (95% CI)								
**ORR**								
A	2.48 (0.66,9.31)	0.17 (0.01,2.15)	1.14 (0.53,2.45)	1.00 (0.31,3.24)	2.67 (0.32,22.60)	0.22 (0.03,1.96)	2.84 (0.59,13.54)	1.63 (0.30,8.75)
0.40 (0.11,1.52)	B	0.07 (0.00,1.20)	0.46 (0.10,2.13)	0.40 (0.07,2.37)	1.08 (0.09,13.32)	1.15 (0.50,2.63)	0.66 (0.08,5.60)	0.75 (0.05,10.78)
6.02 (0.46,77.98)	14.90 (0.83,266.46)	C	6.86 (0.60,79.02)	6.02 (0.36,100.81)	16.07 (0.69,376.81)	17.07 (0.85,343.12)	9.83 (0.56,172.46)	11.14 (0.42,295.89)
0.88 (0.41,1.89)	2.17 (0.47,10.04)	0.15 (0.01,1.68)	D	0.88 (0.22,3.57)	2.34 (0.32,17.22)	2.49 (0.44,14.19)	1.43 (0.32,6.39)	1.62 (0.18,14.46)
1.00 (0.31,3.24)	2.48 (0.42,14.55)	0.17 (0.01,2.78)	1.14 (0.28,4.63)	E	2.67 (0.23,30.57)	2.84 (0.40,20.04)	1.63 (0.21,12.67)	1.85 (0.14,24.85)
0.37 (0.04,3.17)	0.93 (0.08,11.45)	0.06 (0.00,1.46)	0.43 (0.06,3.14)	0.37 (0.03,4.29)	F	1.06 (0.08,14.98)	0.61 (0.16,2.30)	0.69 (0.28,1.70)
4.45 (0.51,38.93)	0.87 (0.38,2.00)	0.06 (0.00,1.18)	0.40 (0.07,2.29)	0.35 (0.05,2.49)	0.94 (0.07,13.28)	G	0.58 (0.06,5.71)	0.65 (0.04,10.67)
0.35 (0.07,1.68)	1.52 (0.18,12.86)	0.10 (0.01,1.79)	0.70 (0.16,3.11)	0.61 (0.08,4.75)	1.64 (0.44,6.14)	1.74 (0.18,17.21)	H	1.13 (0.23,5.61)
0.61 (0.11,3.28)	1.34 (0.09,19.29)	0.09 (0.00,2.39)	0.62 (0.07,5.48)	0.54 (0.04,7.26)	1.44 (0.59,3.55)	1.53 (0.09,25.06)	0.88 (0.18,4.37)	I
0.54 (0.05,5.48)	1.63 (0.10,25.37)	0.11 (0.00,3.09)	0.75 (0.08,7.33)	0.66 (0.05,9.56)	1.76 (0.58,5.31)	1.87 (0.11,32.85)	1.08 (0.19,6.03)	1.22 (0.64,2.32)
0.66 (0.06,7.29)	0.74 (0.04,13.60)	0.05 (0.00,1.61)	0.34 (0.03,4.06)	0.30 (0.02,5.15)	0.80 (0.18,3.47)	0.85 (0.04,17.50)	0.49 (0.07,3.53)	0.55 (0.17,1.77)
0.30 (0.02,3.99)	3.80 (0.76,19.08)	0.26 (0.02,3.88)	1.75 (0.53,5.80)	1.54 (0.34,6.83)	4.10 (0.40,41.96)	4.36 (0.71,26.72)	2.51 (0.37,17.01)	2.84 (0.23,34.36)
1.54 (0.61,3.86)	1.34 (0.59,3.05)	0.09 (0.00,1.80)	0.62 (0.11,3.50)	0.54 (0.08,3.81)	1.44 (0.10,20.34)	1.53 (0.48,4.94)	0.88 (0.09,8.74)	1.00 (0.06,16.34)
1.00 (0.24,4.18)	0.62 (0.03,11.72)	0.04 (0.00,1.38)	0.28 (0.02,3.51)	0.25 (0.01,4.44)	0.67 (0.14,3.08)	0.71 (0.03,15.06)	0.41 (0.05,3.08)	0.46 (0.13,1.60)
0.54 (0.11,2.57)	0.76 (0.04,16.01)	0.05 (0.00,1.86)	0.35 (0.03,4.88)	0.31 (0.02,6.08)	0.82 (0.15,4.60)	0.87 (0.04,20.50)	0.50 (0.06,4.41)	0.57 (0.13,2.48)
0.25 (0.02,3.45)	0.63 (0.03,15.96)	0.04 (0.00,1.80)	0.29 (0.02,5.00)	0.26 (0.01,6.08)	0.68 (0.09,5.17)	0.73 (0.03,20.31)	0.42 (0.04,4.69)	0.47 (0.08,2.90)
0.31 (0.02,4.78)	1.33 (0.08,22.10)	0.09 (0.00,2.67)	0.61 (0.06,6.47)	0.54 (0.03,8.35)	1.44 (0.41,5.05)	1.53 (0.08,28.55)	0.88 (0.14,5.45)	1.00 (0.41,2.40)
**1-year OS rate**								
A	1.14 (0.57,2.29)	0.95 (0.64,1.41)	0.50 (0.16,1.58)	0.69 (0.14,3.27)	1.26 (0.26,6.23)	0.89 (0.37,2.15)	0.58 (0.20,1.72)	0.43 (0.06,2.92)
0.88 (0.44,1.76)	B	0.84 (0.38,1.86)	0.44 (0.11,1.68)	0.60 (0.11,3.32)	0.78 (0.45,1.34)	0.51 (0.14,1.85)	0.38 (0.05,2.89)	**0.09 (0.01,0.78)**
1.05 (0.71,1.55)	1.20 (0.54,2.66)	D	0.52 (0.16,1.77)	0.72 (0.16,3.26)	0.93 (0.35,2.45)	0.61 (0.22,1.68)	0.45 (0.07,2.94)	**0.11 (0.02,0.80)**
2.00 (0.63,6.32)	2.28 (0.59,8.76)	1.91 (0.57,6.43)	E	1.37 (0.20,9.54)	1.77 (0.42,7.57)	1.17 (0.24,5.67)	0.86 (0.09,8.02)	0.21 (0.02,2.15)
1.46 (0.31,6.96)	1.66 (0.30,9.21)	1.39 (0.31,6.31)	0.73 (0.10,5.07)	F	1.29 (0.21,7.79)	0.85 (0.28,2.62)	0.63 (0.21,1.90)	**0.15 (0.04,0.55)**
0.79 (0.16,3.92)	1.29 (0.75,2.22)	1.07 (0.41,2.83)	0.56 (0.13,2.41)	0.77 (0.13,4.66)	G	0.66 (0.16,2.66)	0.48 (0.06,3.99)	0.12 (0.01,1.08)
1.13 (0.47,2.73)	1.96 (0.54,7.09)	1.63 (0.60,4.49)	0.86 (0.18,4.16)	1.18 (0.38,3.63)	1.52 (0.38,6.16)	H	0.74 (0.15,3.57)	**0.18 (0.03,0.99**)
1.71 (0.58,5.06)	2.65 (0.35,20.35)	2.22 (0.34,14.43)	1.16 (0.12,10.85)	1.59 (0.53,4.82)	2.06 (0.25,16.99)	1.36 (0.28,6.58)	I	0.25 (0.13,0.46)
2.33 (0.34,15.77)	**10.83 (1.28,91.52)**	**9.04 (1.25,65.44)**	4.74 (0.47,48.38)	**6.51 (1.81,23.34)**	8.42 (0.93,76.18)	**5.54 (1.01,30.39)**	**4.08 (2.16,7.72)**	K
**9.49 (1.26,71.34)**	**10.54 (1.14,97.62)**	**8.80 (1.10,70.28)**	4.62 (0.42,51.24)	**6.33 (1.52,26.34)**	8.19 (0.83,81.03)	5.39 (0.88,33.13)	**3.97 (1.62,9.75)**	0.97 (0.52,1.83)
**9.24 (1.12,76.48)**	0.83 (0.31,2.21)	0.70 (0.32,1.53)	0.36 (0.10,1.39)	0.50 (0.09,2.75)	0.65 (0.21,1.98)	0.43 (0.12,1.53)	0.31 (0.04,2.40)	0.08 (0.01,0.65)
0.73 (0.37,1.44)	1.34 (0.78,2.31)	1.12 (0.43,2.95)	0.59 (0.14,2.51)	0.81 (0.13,4.86)	1.05 (0.48,2.25)	0.69 (0.17,2.78)	0.51 (0.06,4.17)	0.12 (0.01,1.12)
1.00 (0.39,2.53)	4.24 (0.43,41.88)	3.54 (0.41,30.29)	1.86 (0.16,21.88)	2.55 (0.56,11.69)	3.30 (0.31,34.70)	2.17 (0.33,14.41)	1.60 (0.56,4.55)	0.39 (0.17,0.90)
1.18 (0.49,2.85)	8.22 (0.85,79.14)	6.86 (0.82,57.13)	3.60 (0.31,41.42)	**4.94 (1.12,21.81)**	6.39 (0.62,65.62)	4.20 (0.65,27.09)	**3.10 (1.15,8.34)**	0.76 (0.36,1.62)
3.72 (0.42,32.92)	**8.91 (1.01,78.75)**	7.44 (0.98,56.51)	3.90 (0.37,41.48)	**5.35 (1.38,20.68)**	6.92 (0.73,65.46)	4.55 (0.78,26.44)	**3.36 (1.54,7.29)**	0.82 (0.53,1.28)
**2-year OS rate**								
A	0.78 (0.34,1.75)	1.00 (0.62,1.62)	**0.30 (0.12,0.73)**	1.25 (0.30,5.18)	0.62 (0.14,2.71)	0.80 (0.30,2.14)	1.11 (0.41,2.96)	0.39 (0.08,2.04)
1.29 (0.57,2.91)	B	1.30 (0.50,3.32)	0.39 (0.12,1.29)	1.61 (0.31,8.29)	1.03 (0.58,1.81)	1.43 (0.40,5.11)	0.51 (0.08,3.17)	**0.09 (0.01,0.77)**
1.00 (0.62,1.60)	0.77 (0.30,1.98)	D	**0.30 (0.11,0.82)**	1.25 (0.33,4.75)	0.79 (0.26,2.38)	1.10 (0.47,2.60)	0.39 (0.08,1.89)	**0.07 (0.01,0.48)**
**3.32 (1.37,8.04)**	2.58 (0.78,8.56)	**3.34 (1.22,9.11)**	E	4.16 (0.78,22.15)	2.65 (0.70,9.98)	3.68 (0.98,13.80)	1.31 (0.20,8.47)	0.23 (0.02,2.06)
0.80 (0.19,3.31)	0.62 (0.12,3.18)	0.80 (0.21,3.06)	0.24 (0.05,1.28)	F	0.64 (0.11,3.60)	0.88 (0.32,2.47)	**0.31 (0.14,0.72)**	**0.05 (0.01,0.23)**
1.62 (0.37,7.13)	0.97 (0.55,1.71)	1.26 (0.42,3.78)	0.38 (0.10,1.42)	1.57 (0.28,8.87)	G	1.39 (0.34,5.61)	0.49 (0.07,3.36)	**0.09 (0.01,0.81)**
1.25 (0.47,3.37)	0.70 (0.20,2.51)	0.91 (0.38,2.14)	0.27 (0.07,1.02)	1.13 (0.41,3.15)	0.72 (0.18,2.91)	H	0.36 (0.09,1.33)	**0.06 (0.01,0.36)**
0.90 (0.34,2.41)	1.97 (0.31,12.31)	2.55 (0.53,12.33)	0.76 (0.12,4.95)	**3.18 (1.38,7.31)**	2.02 (0.30,13.78)	2.81 (0.75,10.53)	I	**0.17 (0.05,0.56)**
2.54 (0.49,13.17)	**11.38 (1.29,100.34)**	**14.74 (2.07,105.14)**	4.42 (0.49,40.14)	**18.37 (4.35,77.48)**	**11.70 (1.23,110.88)**	**16.25 (2.78,95.14)**	**5.78 (1.78,18.71)**	K
**14.68 (1.94,110.81)**	4.69 (0.38,58.02)	6.08 (0.59,62.70)	1.82 (0.14,23.12)	**7.57 (1.12,51.28)**	4.82 (0.37,63.51)	6.70 (0.77,58.69)	2.38 (0.43,13.34)	0.41 (0.12,1.45)
6.05 (0.56,65.50)	0.98 (0.34,2.79)	1.26 (0.56,2.87)	0.38 (0.13,1.15)	1.58 (0.33,7.56)	1.00 (0.30,3.31)	1.39 (0.43,4.57)	0.50 (0.08,2.93)	**0.09 (0.01,0.72)**
1.26 (0.65,2.45)	1.32 (0.75,2.34)	1.72 (0.57,5.16)	0.51 (0.14,1.94)	2.14 (0.38,12.09)	1.36 (0.61,3.04)	1.89 (0.47,7.64)	0.67 (0.10,4.58)	0.12 (0.01,1.10)
1.00 (0.37,2.67)	5.00 (0.44,57.04)	6.47 (0.68,61.25)	1.94 (0.17,22.75)	**8.06 (1.32,49.10)**	5.14 (0.42,62.56)	7.13 (0.89,56.94)	2.54 (0.51,12.61)	0.44 (0.15,1.31)
1.71 (0.63,4.60)	5.22 (0.51,53.29)	6.76 (0.81,56.66)	2.03 (0.19,21.28)	**8.42 (1.61,44.00)**	5.36 (0.49,58.63)	**7.45 (1.06,52.13)**	2.65 (0.63,11.06)	0.46 (0.20,1.03)
6.44 (0.65,64.10)	7.82 (0.84,72.98)	**10.13 (1.31,78.23)**	3.04 (0.31,29.61)	**12.62 (2.69,59.26)**	8.04 (0.80,80.51)	**11.17 (1.75,71.40)**	**3.97 (1.08,14.58)**	0.69 (0.39,1.20)
**3-year OS rate**								
A	0.29 (0.08,1.02)	1.13 (0.80,1.58)	**0.32 (0.15,0.72)**	0.71 (0.18,2.75)	0.68 (0.13,3.66)	0.43 (0.11,1.69)	0.65 (0.27,1.57)	0.23 (0.05,1.11)
3.50 (0.98,12.50)	B	**3.94 (1.05,14.72)**	1.13 (0.25,5.10)	2.47 (0.38,15.91)	1.51 (0.93,2.47)	2.26 (0.48,10.66)	0.80 (0.11,6.11)	0.16 (0.01,2.12)
0.89 (0.63,1.25)	**0.25 (0.07,0.95)**	D	**0.29 (0.12,0.69)**	0.63 (0.17,2.34)	0.38 (0.09,1.57)	0.57 (0.25,1.30)	**0.20 (0.04,0.95)**	**0.04 (0.00,0.37)**
**3.09 (1.38,6.88)**	0.88 (0.20,3.97)	**3.47 (1.45,8.30)**	E	2.18 (0.45,10.56)	1.34 (0.27,6.50)	1.99 (0.60,6.59)	0.71 (0.12,4.17)	0.14 (0.01,1.53)
1.41 (0.36,5.49)	0.40 (0.06,2.60)	1.59 (0.43,5.92)	0.46 (0.09,2.22)	F	0.61 (0.09,4.19)	0.91 (0.33,2.55)	**0.32 (0.14,0.73)**	**0.07 (0.01,0.39)**
1.47 (0.27,7.89)	0.66 (0.40,1.08)	2.60 (0.64,10.62)	0.75 (0.15,3.64)	1.64 (0.24,11.20)	G	1.49 (0.29,7.60)	0.53 (0.07,4.28)	0.11 (0.01,1.47)
2.31 (0.59,9.05)	0.44 (0.09,2.09)	1.74 (0.77,3.96)	0.50 (0.15,1.66)	1.10 (0.39,3.06)	0.67 (0.13,3.42)	H	0.36 (0.10,1.32)	**0.07 (0.01,0.56)**
1.55 (0.64,3.77)	1.25 (0.16,9.51)	**4.91 (1.05,23.03)**	1.41 (0.24,8.33)	**3.09 (1.37,6.97)**	1.89 (0.23,15.26)	2.82 (0.76,10.44)	I	**0.20 (0.04,0.97)**
4.37 (0.90,21.24)	6.11 (0.47,79.14)	**24.05 (2.67,216.31)**	6.92 (0.65,73.55)	**15.11 (2.60,87.95)**	9.24 (0.68,125.50)	**13.79 (1.80,105.85)**	**4.89 (1.03,23.34)**	K
**21.37 (2.31,197.35)**	2.96 (0.09,100.24)	11.66 (0.45,305.57)	3.36 (0.11,98.61)	7.33 (0.37,145.80)	4.48 (0.13,156.97)	6.69 (0.28,157.83)	2.37 (0.13,42.18)	0.48 (0.04,5.44)
10.36 (0.39,276.40)	0.57 (0.14,2.32)	**2.23 (1.10,4.49)**	0.64 (0.23,1.76)	1.40 (0.32,6.20)	0.86 (0.19,3.82)	1.28 (0.43,3.75)	0.45 (0.08,2.47)	**0.09 (0.01,0.93)**
**1.98 (1.07,3.65)**	1.12 (0.67,1.88)	**4.41 (1.07,18.18)**	1.27 (0.26,6.23)	2.77 (0.40,19.13)	1.70 (0.83,3.46)	2.53 (0.49,12.99)	0.90 (0.11,7.30)	0.18 (0.01,2.50)
1.00 (0.42,2.39)	3.22 (0.19,55.57)	**12.68 (1.01,158.37)**	3.65 (0.25,52.76)	7.97 (0.92,68.88)	4.87 (0.27,87.71)	7.27 (0.67,79.21)	2.58 (0.35,19.02)	0.53 (0.15,1.83)
3.92 (0.99,15.49)	2.41 (0.16,37.41)	9.51 (0.86,105.09)	2.74 (0.21,35.26)	5.98 (0.80,44.70)	3.66 (0.23,59.15)	5.45 (0.57,52.18)	1.94 (0.31,12.19)	0.40 (0.15,1.05)
11.26 (0.88,143.99)	2.56 (0.18,36.42)	**10.10 (1.01,101.01)**	2.91 (0.25,34.10)	6.35 (0.96,42.09)	3.88 (0.26,57.66)	5.79 (0.67,49.81)	2.06 (0.37,11.34)	**0.42 (0.21,0.84)**

OR (95% CI)								
**ORR**								
A	1.85 (0.18,18.76)	1.52 (0.14,16.79)	3.34 (0.25,44.59)	0.65 (0.26,1.64)	1.00 (0.24,4.18)	1.85 (0.39,8.79)	4.00 (0.29,55.28)	3.25 (0.21,50.55)
0.40 (0.11,1.52)	0.61 (0.04,9.54)	1.35 (0.07,24.78)	0.26 (0.05,1.32)	0.75 (0.33,1.70)	1.62 (0.09,30.61)	1.31 (0.06,27.65)	1.58 (0.06,39.69)	0.75 (0.05,12.47)
6.02 (0.46,77.98)	9.14 (0.32,258.35)	20.11 (0.62,652.30)	3.92 (0.26,59.63)	11.12 (0.55,223.19)	24.08 (0.72,801.66)	19.58 (0.54,712.41)	23.49 (0.55,996.17)	11.19 (0.38,333.91)
0.88 (0.41,1.89)	1.33 (0.14,13.01)	2.93 (0.25,34.88)	0.57 (0.17,1.89)	1.62 (0.29,9.22)	3.51 (0.28,43.32)	2.86 (0.20,39.81)	3.43 (0.20,58.65)	1.63 (0.15,17.24)
1.00 (0.31,3.24)	1.52 (0.10,22.04)	3.34 (0.19,57.48)	0.65 (0.15,2.90)	1.85 (0.26,13.03)	4.00 (0.23,71.05)	3.25 (0.16,64.34)	3.90 (0.16,92.67)	1.86 (0.12,28.86)
0.37 (0.04,3.17)	0.57 (0.19,1.72)	1.25 (0.29,5.44)	0.24 (0.02,2.50)	0.69 (0.05,9.74)	1.50 (0.32,6.92)	1.22 (0.22,6.83)	1.46 (0.19,11.06)	0.70 (0.20,2.45)
4.45 (0.51,38.93)	0.54 (0.03,9.41)	1.18 (0.06,24.28)	0.23 (0.04,1.41)	0.65 (0.20,2.10)	1.41 (0.07,29.96)	1.15 (0.05,26.96)	1.38 (0.05,38.47)	0.66 (0.04,12.27)
0.35 (0.07,1.68)	0.93 (0.17,5.21)	2.05 (0.28,14.77)	0.40 (0.06,2.71)	1.13 (0.11,11.19)	2.45 (0.32,18.50)	1.99 (0.23,17.49)	2.39 (0.21,26.80)	1.14 (0.18,7.06)
0.61 (0.11,3.28)	0.82 (0.43,1.56)	1.81 (0.56,5.77)	0.35 (0.03,4.26)	1.00 (0.06,16.30)	2.16 (0.63,7.46)	1.76 (0.40,7.65)	2.11 (0.34,12.93)	1.00 (0.42,2.42)
0.54 (0.05,5.48)	K	2.20 (0.84,5.79)	0.43 (0.03,5.63)	1.22 (0.07,21.37)	2.64 (0.91,7.59)	2.14 (0.57,8.04)	2.57 (0.47,14.01)	1.22 (0.67,2.23)
0.66 (0.06,7.29)	0.45 (0.17,1.20)	L	0.19 (0.01,3.05)	0.55 (0.03,11.38)	1.20 (0.29,5.02)	0.97 (0.19,5.02)	1.17 (0.17,8.23)	0.56 (0.18,1.74)
0.30 (0.02,3.99)	2.33 (0.18,30.58)	5.13 (0.33,80.24)	M	2.84 (0.46,17.36)	6.14 (0.38,99.29)	4.99 (0.28,90.23)	5.99 (0.27,130.65)	2.86 (0.20,40.14)
1.54 (0.61,3.86)	0.82 (0.05,14.42)	1.81 (0.09,37.21)	0.35 (0.06,2.16)	N	2.16 (0.10,45.90)	1.76 (0.07,41.32)	2.11 (0.08,58.95)	1.01 (0.05,18.80)
1.00 (0.24,4.18)	0.38 (0.13,1.09)	0.84 (0.20,3.50)	0.16 (0.01,2.63)	0.46 (0.02,9.80)	O	0.81 (0.15,4.42)	0.98 (0.13,7.20)	0.46 (0.14,1.57)
0.54 (0.11,2.57)	0.47 (0.12,1.75)	1.03 (0.20,5.29)	0.20 (0.01,3.62)	0.57 (0.02,13.33)	1.23 (0.23,6.69)	P	1.20 (0.14,10.30)	0.57 (0.13,2.44)
0.25 (0.02,3.45)	0.39 (0.07,2.12)	0.86 (0.12,6.03)	0.17 (0.01,3.64)	0.47 (0.02,13.21)	1.02 (0.14,7.56)	0.83 (0.10,7.15)	Q	0.48 (0.08,2.88)
0.31 (0.02,4.78)	0.82 (0.45,1.49)	1.80 (0.57,5.61)	0.35 (0.02,4.92)	0.99 (0.05,18.57)	2.15 (0.64,7.26)	1.75 (0.41,7.48)	2.10 (0.35,12.68)	R
**1-year OS rate**								
A	**0.11 (0.01,0.79)**	**0.11 (0.01,0.90)**	1.37 (0.69,2.71)	1.00 (0.39,2.53)	0.85 (0.35,2.05)	0.27 (0.03,2.38)		
0.88 (0.44,1.76)	**0.09 (0.01,0.88)**	1.20 (0.45,3.19)	0.74 (0.43,1.28)	0.24 (0.02,2.33)	0.12 (0.01,1.17)	**0.11 (0.01,0.99)**		
1.05 (0.71,1.55)	**0.11 (0.01,0.91)**	1.44 (0.65,3.16)	0.89 (0.34,2.34)	0.28 (0.03,2.41)	0.15 (0.02,1.21)	0.13 (0.02,1.02)		
2.00 (0.63,6.32)	0.22 (0.02,2.40)	2.74 (0.72,10.44)	1.70 (0.40,7.24)	0.54 (0.05,6.33)	0.28 (0.02,3.19)	0.26 (0.02,2.72)		
1.46 (0.31,6.96)	**0.16 (0.04,0.66)**	2.00 (0.36,10.99)	1.24 (0.21,7.45)	0.39 (0.09,1.80)	**0.20 (0.05,0.89)**	**0.19 (0.05,0.72)**		
0.79 (0.16,3.92)	0.12 (0.01,1.21)	1.54 (0.51,4.72)	0.96 (0.44,2.06)	0.30 (0.03,3.19)	0.16 (0.02,1.61)	0.14 (0.02,1.37)		
1.13 (0.47,2.73)	0.19 (0.03,1.14)	2.35 (0.65,8.45)	1.45 (0.36,5.89)	0.46 (0.07,3.06)	0.24 (0.04,1.53)	0.22 (0.04,1.28)		
1.71 (0.58,5.06)	0.25 (0.10,0.62)	3.19 (0.42,24.31)	1.97 (0.24,16.25)	0.63 (0.22,1.78)	**0.32 (0.12,0.87)**	**0.30 (0.14,0.65)**		
2.33 (0.34,15.77)	1.03 (0.55,1.93)	**13.00 (1.55,109.35)**	8.05 (0.89,72.86)	**2.55 (1.11,5.85)**	1.32 (0.62,2.81)	1.22 (0.78,1.89)		
**9.49 (1.26,71.34)**	L	**12.65 (1.37,116.66)**	7.84 (0.79,77.50)	2.48 (0.88,7.04)	1.28 (0.48,3.44)	1.18 (0.55,2.56)		
**9.24 (1.12,76.48)**	0.08 (0.01,0.73)	M	0.62 (0.20,1.89)	0.20 (0.02,1.93)	**0.10 (0.01,0.97)**	0.09 (0.01,0.82)		
0.73 (0.37,1.44)	0.13 (0.01,1.26)	1.61 (0.53,4.93)	N	0.32 (0.03,3.33)	0.16 (0.02,1.68)	0.15 (0.02,1.43)		
1.00 (0.39,2.53)	0.40 (0.14,1.14)	5.09 (0.52,50.06)	3.16 (0.30,33.19)	O	0.52 (0.17,1.59)	0.48 (0.19,1.22)		
1.18 (0.49,2.85)	0.78 (0.29,2.09)	**9.87 (1.03,94.58)**	6.11 (0.60,62.76)	1.94 (0.63,5.95)	P	0.92 (0.38,2.22)		
3.72 (0.42,32.92)	0.84 (0.39,1.83)	**10.69 (1.21,94.11)**	6.62 (0.70,62.61)	2.10 (0.82,5.37)	1.08 (0.45,2.61)	R		
**2-year OS rate**								
A	**0.07 (0.01,0.51)**	0.17 (0.02,1.79)	0.79 (0.41,1.54)	1.00 (0.37,2.67)	0.59 (0.22,1.58)	0.16 (0.02,1.54)		
1.29 (0.57,2.91)	0.21 (0.02,2.64)	1.02 (0.36,2.93)	0.76 (0.43,1.33)	0.20 (0.02,2.29)	0.19 (0.02,1.96)	0.13 (0.01,1.19)		
1.00 (0.62,1.60)	0.16 (0.02,1.70)	0.79 (0.35,1.79)	0.58 (0.19,1.75)	0.15 (0.02,1.46)	0.15 (0.02,1.24)	**0.10 (0.01,0.76)**		
**3.32 (1.37,8.04)**	0.55 (0.04,6.96)	2.64 (0.87,7.97)	1.95 (0.52,7.35)	0.52 (0.04,6.05)	0.49 (0.05,5.19)	0.33 (0.03,3.21)		
0.80 (0.19,3.31)	**0.13 (0.02,0.89)**	0.63 (0.13,3.04)	0.47 (0.08,2.65)	**0.12 (0.02,0.76)**	**0.12 (0.02,0.62)**	**0.08 (0.02,0.37)**		
1.62 (0.37,7.13)	0.21 (0.02,2.73)	1.00 (0.30,3.28)	0.73 (0.33,1.64)	0.19 (0.02,2.37)	0.19 (0.02,2.04)	0.12 (0.01,1.25)		
1.25 (0.47,3.37)	0.15 (0.02,1.31)	0.72 (0.22,2.35)	0.53 (0.13,2.14)	0.14 (0.02,1.12)	**0.13 (0.02,0.94)**	**0.09 (0.01,0.57)**		
0.90 (0.34,2.41)	0.42 (0.07,2.35)	2.02 (0.34,11.90)	1.49 (0.22,10.14)	0.39 (0.08,1.96)	0.38 (0.09,1.58)	**0.25 (0.07,0.93)**		
2.54 (0.49,13.17)	2.43 (0.69,8.54)	**11.65 (1.39,97.88)**	8.59 (0.91,81.54)	2.28 (0.76,6.79)	2.18 (0.97,4.92)	1.46 (0.83,2.55)		
**14.68 (1.94,110.81)**	L	4.81 (0.41,56.97)	3.54 (0.27,46.70)	0.94 (0.18,4.97)	0.90 (0.20,4.03)	0.60 (0.15,2.38)		
6.05 (0.56,65.50)	0.21 (0.02,2.47)	M	0.74 (0.22,2.43)	0.20 (0.02,2.14)	0.19 (0.02,1.83)	0.12 (0.01,1.13)		
1.26 (0.65,2.45)	0.28 (0.02,3.72)	1.36 (0.41,4.48)	N	0.27 (0.02,3.23)	0.25 (0.02,2.78)	0.17 (0.02,1.70)		
1.00 (0.37,2.67)	1.06 (0.20,5.64)	5.12 (0.47,55.93)	3.77 (0.31,46.00)	O	0.96 (0.25,3.74)	0.64 (0.19,2.18)		
1.71 (0.63,4.60)	1.11 (0.25,4.98)	5.34 (0.55,52.15)	3.94 (0.36,43.11)	1.04 (0.27,4.08)	P	0.67 (0.25,1.79)		
6.44 (0.65,64.10)	1.67 (0.42,6.61)	8.01 (0.89,72.40)	5.91 (0.59,59.20)	1.57 (0.46,5.34)	1.50 (0.56,4.02)	R		
**3-year OS rate**								
A	**0.05 (0.01,0.43)**	0.10 (0.00,2.57)	**0.51 (0.27,0.93)**	1.00 (0.42,2.39)	0.26 (0.06,1.01)	0.09 (0.01,1.14)		
3.50 (0.98,12.50)	0.34 (0.01,11.43)	1.77 (0.43,7.27)	0.89 (0.53,1.50)	0.31 (0.02,5.36)	0.41 (0.03,6.42)	0.39 (0.03,5.54)		
0.89 (0.63,1.25)	0.09 (0.00,2.25)	**0.45 (0.22,0.91)**	**0.23 (0.06,0.93)**	**0.08 (0.01,0.99)**	0.11 (0.01,1.16)	**0.10 (0.01,0.99)**		
**3.09 (1.38,6.88)**	0.30 (0.01,8.75)	1.56 (0.57,4.28)	0.79 (0.16,3.87)	0.27 (0.02,3.96)	0.37 (0.03,4.70)	0.34 (0.03,4.03)		
1.41 (0.36,5.49)	0.14 (0.01,2.71)	0.71 (0.16,3.17)	0.36 (0.05,2.49)	0.13 (0.01,1.09)	0.17 (0.02,1.25)	0.16 (0.02,1.04)		
1.47 (0.27,7.89)	0.22 (0.01,7.81)	1.17 (0.26,5.21)	0.59 (0.29,1.20)	0.21 (0.01,3.70)	0.27 (0.02,4.43)	0.26 (0.02,3.83)		
2.31 (0.59,9.05)	0.15 (0.01,3.53)	0.78 (0.27,2.30)	0.40 (0.08,2.03)	0.14 (0.01,1.50)	0.18 (0.02,1.76)	0.17 (0.02,1.49)		
1.55 (0.64,3.77)	0.42 (0.02,7.49)	2.21 (0.40,12.04)	1.11 (0.14,9.06)	0.39 (0.05,2.86)	0.52 (0.08,3.25)	0.49 (0.09,2.68)		
4.37 (0.90,21.24)	2.06 (0.18,23.11)	**10.80 (1.08,108.39)**	5.45 (0.40,74.41)	1.90 (0.55,6.59)	2.53 (0.96,6.69)	**2.38 (1.19,4.75)**		
**21.37 (2.31,197.35)**	L	5.24 (0.19,147.88)	2.64 (0.08,92.95)	0.92 (0.06,13.95)	1.23 (0.09,16.60)	1.15 (0.09,14.26)		
10.36 (0.39,276.40)	0.19 (0.01,5.39)	M	0.50 (0.11,2.27)	0.18 (0.01,2.41)	0.23 (0.02,2.86)	0.22 (0.02,2.45)		
**1.98 (1.07,3.65)**	0.38 (0.01,13.29)	1.98 (0.44,8.92)	N	0.35 (0.02,6.29)	0.46 (0.03,7.54)	0.44 (0.03,6.52)		
1.00 (0.42,2.39)	1.09 (0.07,16.48)	5.69 (0.41,78.28)	2.87 (0.16,51.98)	O	1.33 (0.27,6.48)	1.26 (0.30,5.22)		
3.92 (0.99,15.49)	0.82 (0.06,11.04)	4.27 (0.35,52.19)	2.16 (0.13,35.06)	0.75 (0.15,3.65)	P	0.94 (0.29,3.11)		
11.26 (0.88,143.99)	0.87 (0.07,10.69)	4.54 (0.41,50.37)	2.29 (0.15,34.19)	0.80 (0.19,3.31)	1.06 (0.32,3.50)	R		

A, CCRT (cisplatin + etoposide); B, CCRT (carboplatin + paclitaxel); C, CCRT (pemetrexed + carboplatin); *CCRT* Concurrent chemoradiotherapy, *CI* Confidence interval; D, CCRT (pemetrexed + cisplatin); E, CCRT (docetaxel + cisplatin); F, CCRT (S-1 + cisplatin); G, CCRT (mitomycin + vindesine + cisplatin); H, CCRT (cisplatin + vinorelbine); I, CCRT (cisplatin); K, RT; L, CCRT (5-FU); *LA-NSCLC* Locally advanced non-small cell lung cancer; N, CCRT (irinotecan + carboplatin); *NMA* Network meta-analysis; O, CCRT (nedaplatin); *OR* Odds radios; Q, CCRT (paclitaxel); *ORR* Objective response rate, *OS* Overall survival; R, CCRT (carboplatin)

### Network meta-analyses for hematological toxicities of different CCRT regimens

The results of network meta-analyses in hematological toxicities and the network evidence plots were shown in Table 10 and Fig. 2B. The results demonstrated that in terms of hematological toxicities, the incidences of anemia, neutropenia, and thrombocytopenia with CCRT (mitomycin + vindesine + cisplatin) were higher than CCRT (carboplatin + paclitaxel) (OR = 2.94, 95% CI = 1.32–6.55; OR = 53.03, 95% CI = 24.50–114.78; OR = 5.11, 95% CI = 2.28–11.45; respectively). The incidences of neutropenia and febrile neutropenia with CCRT (cisplatin + etoposide) were higher than CCRT (pemetrexed + cisplatin) (OR = 1.79, 95% CI = 1.20–2.66; OR = 2.42, 95% CI = 1.08–5.40; respectively). The incidences of neutropenia and thrombocytopenia with CCRT (irinotecan + carboplatin) were higher than CCRT (carboplatin + paclitaxel) (OR = 3.88, 95% CI = 2.37–6.37; OR = 2.40, 95% CI = 1.01–5.71; respectively). The incidence of leukopenia with CCRT (cisplatin + vinorelbine) was higher than CCRT (S-1 + cisplatin) and CCRT (cisplatin) (OR = 5.34, 95% CI = 1.23–23.14; OR = 7.41, 95% CI = 1.14–48.39; respectively). The incidence of leukopenia with CCRT (carboplatin) was higher than CCRT (S-1 + cisplatin), CCRT (cisplatin), and RT (OR = 25.48, 95% CI = 2.70–240.60; OR = 35.35, 95% CI = 5.23–239.20, OR = 38.74, 95% CI = 8.50–176.58; respectively). The incidence of leukopenia with CCRT (nedaplatin) was higher than CCRT (cisplatin) and RT (OR = 22.60, 95% CI = 1.74–293.03; OR = 24.76, 95% CI = 2.53–242.76; respectively). The incidence of neutropenia with CCRT (etoposide + cisplatin) was higher than CCRT (paclitaxel + carboplatin) (OR = 3.28, 95% CI = 1.52–7.09). The incidence of neutropenia with CCRT (irinotecan + carboplatin) was higher than CCRT (etoposide + cisplatin) (OR = 16.15, 95% CI = 5.43–48.06). The incidence of neutropenia with CCRT (mitomycin + vindesine + cisplatin) was higher than CCRT (pemetrexed + cisplatin), CCRT (docetaxel + cisplatin), and CCRT (irinotecan + carboplatin) (OR = 28.85, 95% CI = 9.03–92.14; OR = 173.39, 95% CI = 7.49–4015.10; OR = 13.66, 95% CI = 5.46–34.17; respectively). The incidence of febrile neutropenia with CCRT (cisplatin + vinorelbine) was higher than CCRT (cisplatin + etoposide) (OR = 6.91, 95% CI = 1.39–34.42).

**Table 10:** Network meta-analyses for toxicities of different CCRT regimens in the treatment of LA-NSCLC

OR (95% CI)								
**Anemia**								
A	0.95 (0.26,3.52)	0.68 (0.35,1.33)	0.91 (0.06,14.90)	1.12 (0.10,12.92)	2.79 (0.60,12.96)	1.28 (0.33,5.03)	1.12 (0.07,18.10)	0.97 (0.05,19.02)
1.05 (0.28,3.90)	B	0.72 (0.16,3.11)	0.95 (0.04,20.98)	1.18 (0.07,18.88)	**2.94 (1.32,6.55)**	1.35 (0.20,8.95)	1.18 (0.05,25.53)	1.02 (0.04,26.36)
1.47 (0.75,2.86)	1.40 (0.32,6.07)	D	1.33 (0.08,23.67)	1.65 (0.16,17.29)	4.10 (0.77,21.86)	1.88 (0.57,6.21)	1.65 (0.11,24.52)	1.43 (0.08,25.90)
1.10 (0.07,18.10)	1.05 (0.05,23.02)	0.75 (0.04,13.33)	E	1.24 (0.03,50.82)	3.07 (0.13,74.85)	1.41 (0.06,31.79)	1.24 (0.02,63.97)	1.07 (0.02,63.58)
0.89 (0.08,10.21)	0.85 (0.05,13.48)	0.61 (0.06,6.34)	0.81 (0.02,33.07)	F	2.48 (0.14,44.35)	1.14 (0.15,8.60)	1.00 (0.26,3.78)	0.86 (0.16,4.73)
0.36 (0.08,1.66)	**0.34 (0.15,0.76)**	0.24 (0.05,1.30)	0.33 (0.01,7.92)	0.40 (0.02,7.21)	G	0.46 (0.06,3.59)	0.40 (0.02,9.64)	0.35 (0.01,9.90)
0.78 (0.20,3.07)	0.74 (0.11,4.93)	0.53 (0.16,1.76)	0.71 (0.03,15.97)	0.88 (0.12,6.64)	2.18 (0.28,17.04)	H	0.88 (0.08,9.87)	0.76 (0.05,10.65)
0.89 (0.06,14.32)	0.85 (0.04,18.24)	0.61 (0.04,8.99)	0.81 (0.02,41.65)	1.00 (0.26,3.78)	2.48 (0.10,59.36)	1.14 (0.10,12.80)	I	0.86 (0.30,2.50)
1.03 (0.05,20.14)	0.98 (0.04,25.22)	0.70 (0.04,12.72)	0.93 (0.02,55.44)	1.16 (0.21,6.34)	2.87 (0.10,81.61)	1.32 (0.09,18.49)	1.16 (0.40,3.34)	K
1.01 (0.06,16.40)	0.96 (0.04,20.88)	0.69 (0.04,12.08)	0.92 (0.02,47.59)	1.14 (0.03,46.18)	2.82 (0.12,67.92)	1.29 (0.06,28.83)	1.14 (0.02,58.15)	0.98 (0.02,57.82)
1.00 (0.25,4.02)	0.68 (0.28,1.64)	0.49 (0.09,2.69)	0.65 (0.03,16.08)	0.80 (0.04,14.67)	1.99 (0.60,6.55)	0.91 (0.11,7.37)	0.80 (0.03,19.59)	0.69 (0.02,20.09)
0.71 (0.15,3.46)	0.33 (0.01,11.35)	0.24 (0.01,5.91)	0.32 (0.00,23.64)	0.39 (0.04,3.52)	0.98 (0.03,36.46)	0.45 (0.02,8.82)	0.39 (0.07,2.24)	0.34 (0.09,1.35)
**Leukopenia**								
A	0.68 (0.23,2.00)	0.34 (0.04,3.22)	1.81 (0.32,10.03)	0.24 (0.02,3.09)	0.22 (0.01,3.63)	0.85 (0.26,2.76)	5.50 (0.15,203.01)	8.61 (0.36,207.28)
1.47 (0.50,4.35)	D	0.50 (0.07,3.61)	2.66 (0.70,10.09)	0.36 (0.04,3.58)	0.33 (0.02,4.32)	1.25 (0.25,6.19)	8.12 (0.26,253.90)	12.70 (0.64,253.22)
2.96 (0.31,28.23)	2.01 (0.28,14.54)	F	**5.34 (1.23,23.14)**	0.72 (0.22,2.33)	0.66 (0.13,3.43)	2.50 (0.20,31.93)	16.29 (0.97,272.60)	**25.48 (2.70,240.60)**
0.55 (0.10,3.08)	0.38 (0.10,1.42)	**0.19 (0.04,0.81)**	H	**0.13 (0.02,0.88)**	0.12 (0.01,1.12)	0.47 (0.06,3.76)	3.05 (0.13,72.97)	4.77 (0.33,69.60)
4.11 (0.32,52.11)	2.78 (0.28,27.78)	1.39 (0.43,4.48)	**7.41 (1.14,48.39)**	I	0.91 (0.28,2.92)	3.47 (0.21,57.22)	**22.60 (1.74,293.03)**	**35.35 (5.23,239.20)**
4.50 (0.28,73.57)	3.05 (0.23,40.17)	1.52 (0.29,7.93)	8.12 (0.89,73.87)	1.10 (0.34,3.51)	K	3.80 (0.18,79.06)	**24.76 (2.53,242.76)**	**38.74 (8.50,176.58)**
1.18 (0.36,3.86)	0.80 (0.16,3.98)	0.40 (0.03,5.10)	2.14 (0.27,17.15)	0.29 (0.02,4.75)	0.26 (0.01,5.46)	M	6.51 (0.15,290.07)	10.19 (0.34,303.31)
0.18 (0.00,6.70)	0.12 (0.00,3.85)	0.06 (0.00,1.03)	0.33 (0.01,7.85)	**0.04 (0.00,0.57)**	**0.04 (0.00,0.40)**	0.15 (0.00,6.84)	O	1.56 (0.10,24.24)
0.12 (0.00,2.80)	0.08 (0.00,1.57)	**0.04 (0.00,0.37)**	0.21 (0.01,3.06)	**0.03 (0.00,0.19)**	**0.03 (0.01,0.12)**	0.10 (0.00,2.92)	0.64 (0.04,9.91)	R
**Neutropenia**								
A	**0.30 (0.14,0.66)**	**0.56 (0.38,0.83)**	0.09 (0.00,1.77)	0.05 (0.00,1.26)	**16.15 (5.43,48.06)**			
**3.28 (1.52,7.09)**	B	1.84 (0.77,4.38)	0.31 (0.01,6.43)	**53.03 (24.50,114.78)**	**3.88 (2.37,6.37)**			
**1.79 (1.20,2.66)**	0.54 (0.23,1.29)	D	0.17 (0.01,3.26)	**28.85 (9.03,92.14)**	2.11 (0.78,5.73)			
10.74 (0.56,204.50)	3.27 (0.16,68.76)	6.01 (0.31,117.62)	E	**173.39 (7.49,4015.10)**	12.70 (0.58,277.83)			
20.73 (0.80,539.92)	**0.02 (0.01,0.04)**	**0.03 (0.01,0.11)**	**0.01 (0.00,0.13)**	G	**0.07 (0.03,0.18)**			
**0.06 (0.02,0.18)**	**0.26 (0.16,0.42)**	0.47 (0.17,1.28)	0.08 (0.00,1.72)	**13.66 (5.46,34.17)**	N			
**Thrombocytopenia**								
A	0.54 (0.15,1.94)	0.75 (0.37,1.50)	0.30 (0.01,7.46)	0.72 (0.03,20.13)	1.72 (0.04,81.13)	2.75 (0.61,12.51)	0.81 (0.14,4.87)	0.63 (0.02,20.47)
1.85 (0.52,6.67)	B	1.38 (0.32,5.94)	0.55 (0.02,17.68)	1.33 (0.04,47.33)	**5.11 (2.28,11.45)**	1.51 (0.17,13.62)	1.17 (0.03,47.69)	1.84 (0.03,114.52)
1.34 (0.67,2.70)	0.72 (0.17,3.11)	D	0.40 (0.01,10.79)	0.96 (0.04,25.08)	3.69 (0.70,19.56)	1.09 (0.21,5.67)	0.85 (0.03,25.59)	1.33 (0.02,71.90)
3.36 (0.13,84.46)	1.81 (0.06,58.16)	2.51 (0.09,67.83)	E	2.41 (0.02,249.13)	9.26 (0.26,325.90)	2.73 (0.07,109.10)	2.12 (0.02,243.56)	3.33 (0.02,536.74)
1.40 (0.05,39.22)	0.75 (0.02,26.80)	1.04 (0.04,27.15)	0.41 (0.00,42.89)	F	3.84 (0.10,149.80)	1.13 (0.07,18.90)	0.88 (0.33,2.37)	1.38 (0.01,239.39)
0.58 (0.01,27.35)	**0.20 (0.09,0.44)**	0.27 (0.05,1.43)	0.11 (0.00,3.80)	0.26 (0.01,10.14)	G	0.29 (0.03,3.08)	0.23 (0.01,10.19)	0.36 (0.01,24.25)
0.36 (0.08,1.65)	0.66 (0.07,6.01)	0.92 (0.18,4.78)	0.37 (0.01,14.63)	0.88 (0.05,14.72)	3.39 (0.32,35.41)	H	0.78 (0.04,15.35)	1.22 (0.02,91.63)
1.23 (0.21,7.40)	0.85 (0.02,34.83)	1.18 (0.04,35.72)	0.47 (0.00,54.08)	1.14 (0.42,3.06)	4.36 (0.10,194.02)	1.29 (0.07,25.42)	I	1.57 (0.01,298.72)
1.59 (0.05,51.44)	0.54 (0.01,33.98)	0.75 (0.01,40.78)	0.30 (0.00,48.41)	0.72 (0.00,125.42)	2.78 (0.04,187.65)	0.82 (0.01,61.63)	0.64 (0.00,121.36)	M
1.01 (0.02,51.45)	**0.42 (0.18,0.99)**	0.58 (0.11,3.14)	0.23 (0.01,8.19)	0.55 (0.01,21.86)	2.13 (0.65,6.95)	0.63 (0.06,6.68)	0.49 (0.01,21.94)	0.76 (0.01,52.17)
**Febrile neutropenia**								
A	0.53 (0.15,1.94)	**0.41 (0.19,0.93)**	3.14 (0.56,17.55)	**6.91 (1.39,34.42)**	0.98 (0.18,5.40)			
1.87 (0.52,6.80)	B	-	-	-	-			
**2.42 (1.08,5.40)**	-	D	-	-	-			
0.32 (0.06,1.78)	-	-	E	-	-			
**0.14 (0.03,0.72)**	-	-	-	H	-			
1.02 (0.19,5.61)	-	-	-	-	N			
**Pneumonia**								
A	2.26 (0.89,5.75)	1.23 (0.05,27.71)	0.82 (0.27,2.47)	6.00 (0.70,51.69)	0.37 (0.01,11.95)	0.70 (0.05,9.80)	**5.65 (1.82,17.56)**	0.89 (0.04,18.04)
0.44 (0.17,1.13)	B	0.54 (0.02,14.08)	0.36 (0.09,1.54)	2.66 (0.25,27.80)	0.16 (0.00,5.99)	**2.50 (1.32,4.75)**	0.39 (0.02,9.21)	0.11 (0.00,6.18)
0.81 (0.04,18.30)	1.84 (0.07,47.42)	C	0.67 (0.04,12.27)	4.88 (0.11,215.04)	0.30 (0.00,24.39)	4.59 (0.17,126.33)	0.72 (0.01,41.13)	0.20 (0.00,23.40)
1.22 (0.40,3.67)	2.75 (0.65,11.69)	1.50 (0.08,27.61)	D	7.31 (0.65,82.23)	0.45 (0.02,12.19)	**6.89 (1.42,33.51)**	1.09 (0.07,17.84)	0.29 (0.01,13.05)
0.17 (0.02,1.44)	0.38 (0.04,3.94)	0.21 (0.00,9.05)	0.14 (0.01,1.54)	E	0.06 (0.00,3.67)	0.94 (0.08,10.74)	0.15 (0.00,6.00)	0.04 (0.00,3.61)
2.74 (0.08,89.67)	6.19 (0.17,229.02)	3.37 (0.04,277.01)	2.25 (0.08,61.54)	16.43 (0.27,991.21)	F	15.48 (0.40,606.42)	2.44 (0.42,14.36)	0.66 (0.10,4.22)
1.42 (0.10,19.81)	0.40 (0.21,0.76)	0.22 (0.01,5.99)	**0.15 (0.03,0.71)**	1.06 (0.09,12.10)	0.06 (0.00,2.53)	G	0.16 (0.01,3.92)	0.04 (0.00,2.60)
**0.18 (0.06,0.55)**	2.53 (0.11,59.03)	1.38 (0.02,78.30)	0.92 (0.06,15.09)	6.73 (0.17,271.71)	0.41 (0.07,2.41)	6.34 (0.25,157.58)	H	0.27 (0.02,3.51)
1.12 (0.06,22.68)	9.36 (0.16,541.63)	5.10 (0.04,608.54)	3.40 (0.08,150.85)	24.87 (0.28,2233.88)	1.51 (0.24,9.66)	23.42 (0.38,1425.39)	3.70 (0.28,47.99)	I
4.14 (0.08,215.04)	**17.61 (1.62,191.58)**	9.60 (0.21,433.60)	6.40 (0.55,74.69)	**46.79 (2.16,1013.71)**	2.85 (0.05,175.65)	**44.08 (3.72,521.75)**	6.96 (0.17,287.98)	1.88 (0.02,172.53)
7.80 (0.87,70.10)	10.53 (0.17,643.87)	5.74 (0.05,717.47)	3.82 (0.08,180.01)	27.97 (0.30,2641.44)	1.70 (0.24,12.24)	26.35 (0.41,1693.33)	4.16 (0.29,58.88)	1.12 (0.57,2.20)
4.66 (0.08,256.01)	1.06 (0.34,3.33)	0.58 (0.02,13.90)	0.38 (0.11,1.39)	2.80 (0.29,26.72)	0.17 (0.00,5.95)	2.64 (0.71,9.84)	0.42 (0.02,9.07)	0.11 (0.00,6.19)
**0.47 (0.24,0.91)**	0.83 (0.41,1.68)	0.45 (0.02,12.60)	0.30 (0.06,1.51)	2.20 (0.19,25.59)	0.13 (0.00,5.32)	2.08 (0.80,5.39)	0.33 (0.01,8.26)	0.09 (0.00,5.45)
1.00 (0.32,3.11)	4.35 (0.04,483.31)	2.37 (0.01,497.48)	1.58 (0.02,139.94)	11.55 (0.07,1884.73)	0.70 (0.03,14.51)	10.88 (0.09,1263.18)	1.72 (0.05,57.25)	0.46 (0.04,5.08)
0.37 (0.11,1.19)	7.15 (0.10,519.42)	3.90 (0.03,564.68)	2.60 (0.05,146.85)	19.00 (0.17,2097.30)	1.16 (0.11,11.65)	17.90 (0.23,1363.33)	2.82 (0.15,51.86)	0.76 (0.19,3.03)
1.93 (0.02,194.87)	11.15 (0.13,967.03)	6.07 (0.04,1026.53)	4.05 (0.06,276.30)	29.62 (0.23,3845.61)	1.80 (0.13,24.88)	27.90 (0.31,2533.45)	4.40 (0.19,104.40)	1.19 (0.19,7.64)
3.17 (0.05,207.45)	5.75 (0.09,388.48)	3.13 (0.02,426.63)	2.09 (0.04,109.33)	15.27 (0.15,1578.86)	0.93 (0.11,8.17)	14.38 (0.20,1020.49)	2.27 (0.14,37.45)	0.61 (0.20,1.91)
**Esophagitis**								
A	1.06 (0.53,2.12)	1.51 (0.08,29.70)	1.01 (0.54,1.88)	0.90 (0.17,4.70)	3.02 (0.08,119.96)	0.63 (0.06,6.23)	0.55 (0.16,1.89)	3.42 (0.32,36.99)
0.95 (0.47,1.90)	B	1.43 (0.07,30.45)	0.95 (0.38,2.42)	0.86 (0.14,5.12)	2.86 (0.07,121.16)	0.52 (0.19,1.44)	3.24 (0.27,38.66)	1.37 (0.02,85.32)
0.66 (0.03,13.01)	0.70 (0.03,14.88)	C	0.67 (0.04,12.27)	0.60 (0.02,17.99)	2.00 (0.02,209.72)	0.36 (0.01,9.13)	2.27 (0.06,92.51)	0.96 (0.01,137.76)
0.99 (0.53,1.85)	1.05 (0.41,2.66)	1.50 (0.08,27.60)	D	0.90 (0.15,5.22)	3.00 (0.08,112.96)	0.54 (0.14,2.17)	3.40 (0.34,33.80)	1.44 (0.03,80.44)
1.11 (0.21,5.75)	1.17 (0.20,6.99)	1.67 (0.06,50.29)	1.11 (0.19,6.49)	E	3.34 (0.06,188.73)	0.61 (0.08,4.76)	3.79 (0.21,68.49)	1.60 (0.02,129.61)
0.33 (0.01,13.15)	0.35 (0.01,14.83)	0.50 (0.00,52.51)	0.33 (0.01,12.57)	0.30 (0.01,16.91)	F	0.18 (0.00,8.83)	1.13 (0.07,18.88)	0.48 (0.08,2.74)
1.58 (0.16,15.63)	1.92 (0.69,5.35)	2.75 (0.11,69.20)	1.84 (0.46,7.32)	1.65 (0.21,12.91)	5.50 (0.11,267.40)	G	6.24 (0.43,91.10)	2.64 (0.04,185.99)
1.82 (0.53,6.26)	0.31 (0.03,3.68)	0.44 (0.01,18.02)	0.29 (0.03,2.93)	0.26 (0.01,4.77)	0.88 (0.05,14.69)	0.16 (0.01,2.34)	H	0.42 (0.02,11.56)
0.29 (0.03,3.15)	0.73 (0.01,45.38)	1.04 (0.01,149.90)	0.70 (0.01,38.91)	0.62 (0.01,50.46)	2.08 (0.36,11.91)	0.38 (0.01,26.69)	2.36 (0.09,64.58)	I
0.69 (0.01,40.50)	1.22 (0.02,80.69)	1.75 (0.01,263.89)	1.16 (0.02,69.29)	1.04 (0.01,89.41)	3.49 (0.53,22.92)	0.63 (0.01,47.39)	3.96 (0.13,116.56)	1.67 (0.82,3.41)
1.16 (0.02,72.08)	0.30 (0.00,24.12)	0.43 (0.00,76.43)	0.29 (0.00,20.82)	0.26 (0.00,26.43)	0.85 (0.09,8.46)	0.16 (0.00,14.09)	0.97 (0.03,36.44)	0.41 (0.09,1.82)
0.28 (0.00,21.61)	**3.96 (1.20,13.03)**	5.67 (0.25,129.81)	**3.78 (1.20,11.94)**	3.39 (0.50,22.91)	11.32 (0.25,509.73)	2.06 (0.43,9.88)	12.84 (0.98,167.58)	5.43 (0.08,356.96)
**3.75 (1.43,9.87)**	**3.90 (1.07,14.28)**	5.58 (0.20,154.69)	3.72 (0.75,18.39)	3.34 (0.37,30.42)	11.15 (0.21,588.13)	2.03 (0.39,10.57)	12.65 (0.77,207.56)	5.35 (0.07,406.22)
1.00 (0.11,8.74)	0.60 (0.01,54.68)	0.85 (0.00,169.97)	0.57 (0.01,47.32)	0.51 (0.00,59.52)	1.71 (0.14,21.36)	0.31 (0.00,31.83)	1.93 (0.04,84.75)	0.82 (0.13,5.10)
3.69 (0.85,16.09)	1.03 (0.01,78.17)	1.48 (0.01,250.11)	0.98 (0.01,67.36)	0.88 (0.01,85.95)	2.95 (0.34,25.81)	0.54 (0.01,45.73)	3.35 (0.10,116.49)	1.42 (0.39,5.14)
0.56 (0.01,49.05)	1.29 (0.01,203.25)	1.84 (0.01,587.33)	1.23 (0.01,177.79)	1.10 (0.01,215.83)	3.67 (0.12,111.03)	0.67 (0.00,116.92)	4.17 (0.05,345.47)	1.76 (0.09,32.98)
0.98 (0.01,69.96)	0.66 (0.01,48.05)	0.94 (0.01,154.59)	0.63 (0.01,41.37)	0.56 (0.01,52.94)	1.88 (0.23,15.32)	0.34 (0.00,28.14)	2.13 (0.06,71.07)	0.90 (0.28,2.90)
**Nausea/vomiting**								
A	0.34 (0.03,3.89)	0.79 (0.42,1.46)	1.85 (0.16,21.04)	0.51 (0.03,9.91)	0.28 (0.01,12.23)	3.85 (0.28,53.21)	0.27 (0.03,2.95)	0.42 (0.02,10.44)
2.97 (0.26,34.22)	B	2.33 (0.19,29.04)	5.48 (0.17,172.54)	1.52 (0.03,70.79)	**11.41 (4.38,29.77)**	0.81 (0.03,24.57)	1.24 (0.02,70.57)	0.94 (0.02,56.26)
1.27 (0.69,2.36)	0.43 (0.03,5.35)	D	2.35 (0.19,28.92)	0.65 (0.04,11.82)	4.90 (0.33,72.75)	0.35 (0.03,3.46)	0.53 (0.02,12.51)	0.40 (0.02,10.12)
0.54 (0.05,6.15)	0.18 (0.01,5.74)	0.43 (0.03,5.22)	E	0.28 (0.01,12.79)	2.08 (0.06,74.61)	0.15 (0.00,4.44)	0.23 (0.00,12.76)	0.17 (0.00,10.17)
1.96 (0.10,37.90)	0.66 (0.01,30.76)	1.54 (0.08,27.91)	3.62 (0.08,167.21)	F	7.52 (0.14,394.95)	0.53 (0.09,3.14)	0.82 (0.23,2.86)	0.62 (0.15,2.54)
3.51 (0.08,150.97)	**0.09 (0.03,0.23)**	0.20 (0.01,3.03)	0.48 (0.01,17.23)	0.13 (0.00,6.98)	G	0.07 (0.00,2.46)	0.11 (0.00,6.92)	0.08 (0.00,5.51)
0.26 (0.02,3.59)	1.23 (0.04,37.40)	2.88 (0.29,28.60)	6.77 (0.23,203.07)	1.87 (0.32,11.00)	14.08 (0.41,487.14)	H	1.53 (0.18,13.38)	1.16 (0.12,11.15)
3.66 (0.34,39.49)	0.81 (0.01,45.88)	1.88 (0.08,44.19)	4.42 (0.08,249.47)	1.22 (0.35,4.28)	9.20 (0.14,585.70)	0.65 (0.07,5.71)	I	0.76 (0.39,1.46)
2.39 (0.10,59.69)	1.07 (0.02,64.00)	2.49 (0.10,62.56)	5.85 (0.10,348.10)	1.62 (0.39,6.66)	12.17 (0.18,816.01)	0.86 (0.09,8.33)	1.32 (0.68,2.56)	K
3.16 (0.12,84.40)	0.43 (0.01,32.31)	1.00 (0.03,33.42)	2.35 (0.03,175.80)	0.65 (0.09,4.70)	4.90 (0.06,409.61)	0.35 (0.02,4.94)	0.53 (0.12,2.46)	0.40 (0.10,1.60)
1.27 (0.04,44.88)	0.18 (0.01,2.35)	0.42 (0.15,1.15)	0.98 (0.08,12.71)	0.27 (0.01,5.86)	2.04 (0.13,31.85)	0.15 (0.01,1.79)	0.22 (0.01,6.12)	0.17 (0.01,4.93)
0.53 (0.24,1.19)	0.71 (0.22,2.29)	1.65 (0.10,26.68)	3.89 (0.10,148.48)	1.08 (0.02,59.78)	**8.10 (1.78,36.76)**	0.57 (0.02,21.19)	0.88 (0.01,59.11)	0.67 (0.01,47.02)
1.00 (0.18,5.63)	0.10 (0.00,8.06)	0.24 (0.01,8.42)	0.56 (0.01,43.84)	0.16 (0.02,1.24)	1.17 (0.01,102.02)	0.08 (0.01,1.27)	**0.13 (0.02,0.66)**	**0.10 (0.02,0.44)**
2.10 (0.14,31.66)	1.61 (0.02,143.47)	3.76 (0.09,153.96)	8.84 (0.10,780.85)	2.45 (0.24,24.89)	18.40 (0.19,1811.86)	1.31 (0.07,24.19)	2.00 (0.28,14.10)	1.51 (0.24,9.51)

OR (95% CI)								
**Anemia**								
A	0.99 (0.06,16.05)	1.00 (0.25,4.02)	1.40 (0.29,6.80)					
1.05 (0.28,3.90)	1.04 (0.05,22.62)	1.48 (0.61,3.56)	3.01 (0.09,102.86)					
1.47 (0.75,2.86)	1.45 (0.08,25.50)	2.06 (0.37,11.43)	4.20 (0.17,104.25)					
1.10 (0.07,18.10)	1.09 (0.02,56.59)	1.55 (0.06,38.42)	3.15 (0.04,235.05)					
0.89 (0.08,10.21)	0.88 (0.02,35.73)	1.25 (0.07,22.81)	2.54 (0.28,22.75)					
0.36 (0.08,1.66)	0.35 (0.01,8.54)	0.50 (0.15,1.66)	1.03 (0.03,38.34)					
0.78 (0.20,3.07)	0.77 (0.03,17.23)	1.10 (0.14,8.85)	2.23 (0.11,44.06)					
0.89 (0.06,14.32)	0.88 (0.02,45.03)	1.25 (0.05,30.47)	2.54 (0.45,14.53)					
1.03 (0.05,20.14)	1.02 (0.02,59.95)	1.44 (0.05,41.85)	2.94 (0.74,11.73)					
1.01 (0.06,16.40)	M	1.42 (0.06,34.86)	2.89 (0.04,213.86)					
1.00 (0.25,4.02)	0.71 (0.03,17.35)	N	2.04 (0.05,77.68)					
0.71 (0.15,3.46)	0.35 (0.00,25.58)	0.49 (0.01,18.67)	R					
**Leukopenia**								
A								
1.47 (0.50,4.35)								
2.96 (0.31,28.23)								
0.55 (0.10,3.08)								
4.11 (0.32,52.11)								
4.50 (0.28,73.57)								
1.18 (0.36,3.86)								
0.18 (0.00,6.70)								
0.12 (0.00,2.80)								
**Neutropenia**								
A								
**3.28 (1.52,7.09)**								
**1.79 (1.20,2.66)**								
10.74 (0.56,204.50)								
20.73 (0.80,539.92)								
**0.06 (0.02,0.18)**								
**Thrombocytopenia**								
A	0.99 (0.02,50.39)							
1.85 (0.52,6.67)	**2.40 (1.01,5.71)**							
1.34 (0.67,2.70)	1.74 (0.32,9.47)							
3.36 (0.13,84.46)	4.36 (0.12,155.32)							
1.40 (0.05,39.22)	1.81 (0.05,71.37)							
0.58 (0.01,27.35)	0.47 (0.14,1.54)							
0.36 (0.08,1.65)	1.59 (0.15,17.00)							
1.23 (0.21,7.40)	2.05 (0.05,92.39)							
1.59 (0.05,51.44)	1.31 (0.02,89.25)							
1.01 (0.02,51.45)	N							
**Febrile neutropenia**								
A								
1.87 (0.52,6.80)								
**2.42 (1.08,5.40)**								
0.32 (0.06,1.78)								
**0.14 (0.03,0.72)**								
1.02 (0.19,5.61)								
**Pneumonia**								
A	0.24 (0.00,12.52)	0.13 (0.01,1.15)	0.21 (0.00,11.78)	**2.14 (1.10,4.16)**	1.00 (0.32,3.11)	2.72 (0.84,8.79)	0.52 (0.01,52.54)	0.32 (0.00,20.69)
0.44 (0.17,1.13)	**0.06 (0.01,0.62)**	0.09 (0.00,5.81)	0.95 (0.30,2.99)	1.21 (0.59,2.44)	0.23 (0.00,25.55)	0.14 (0.00,10.16)	0.09 (0.00,7.78)	0.17 (0.00,11.76)
0.81 (0.04,18.30)	0.10 (0.00,4.71)	0.17 (0.00,21.80)	1.74 (0.07,42.00)	2.21 (0.08,61.67)	0.42 (0.00,88.60)	0.26 (0.00,37.20)	0.16 (0.00,27.82)	0.32 (0.00,43.50)
1.22 (0.40,3.67)	0.16 (0.01,1.82)	0.26 (0.01,12.31)	2.61 (0.72,9.46)	3.32 (0.66,16.60)	0.63 (0.01,56.08)	0.38 (0.01,21.77)	0.25 (0.00,16.85)	0.48 (0.01,25.09)
0.17 (0.02,1.44)	**0.02 (0.00,0.46)**	0.04 (0.00,3.38)	0.36 (0.04,3.40)	0.45 (0.04,5.27)	0.09 (0.00,14.12)	0.05 (0.00,5.81)	0.03 (0.00,4.38)	0.07 (0.00,6.77)
2.74 (0.08,89.67)	0.35 (0.01,21.66)	0.59 (0.08,4.22)	5.86 (0.17,204.25)	7.46 (0.19,295.63)	1.42 (0.07,29.35)	0.86 (0.09,8.71)	0.55 (0.04,7.66)	1.08 (0.12,9.46)
1.42 (0.10,19.81)	**0.02 (0.00,0.27)**	0.04 (0.00,2.44)	0.38 (0.10,1.41)	0.48 (0.19,1.25)	0.09 (0.00,10.66)	0.06 (0.00,4.26)	0.04 (0.00,3.25)	0.07 (0.00,4.93)
**0.18 (0.06,0.55)**	0.14 (0.00,5.95)	0.24 (0.02,3.41)	2.40 (0.11,52.17)	3.05 (0.12,76.95)	0.58 (0.02,19.41)	0.35 (0.02,6.50)	0.23 (0.01,5.39)	0.44 (0.03,7.27)
1.12 (0.06,22.68)	0.53 (0.01,48.72)	0.89 (0.45,1.74)	8.87 (0.16,486.31)	11.28 (0.18,693.98)	2.15 (0.20,23.56)	1.31 (0.33,5.19)	0.84 (0.13,5.38)	1.63 (0.52,5.06)
4.14 (0.08,215.04)	J	1.67 (0.02,161.18)	**16.68 (1.68,165.50)**	**21.23 (1.76,255.84)**	4.05 (0.02,672.71)	2.46 (0.02,277.28)	1.58 (0.01,209.07)	3.06 (0.03,323.21)
7.80 (0.87,70.10)	0.60 (0.01,57.60)	K	9.97 (0.17,578.52)	12.69 (0.20,824.32)	2.42 (0.24,24.06)	1.47 (0.44,4.90)	0.94 (0.17,5.34)	1.83 (0.73,4.57)
4.66 (0.08,256.01)	**0.06 (0.01,0.59)**	0.10 (0.00,5.82)	M	1.27 (0.33,4.90)	0.24 (0.00,25.77)	0.15 (0.00,10.20)	0.09 (0.00,7.83)	0.18 (0.00,11.79)
**0.47 (0.24,0.91)**	**0.05 (0.00,0.57)**	0.08 (0.00,5.12)	0.79 (0.20,3.03)	N	0.19 (0.00,22.35)	0.12 (0.00,8.93)	0.07 (0.00,6.82)	0.14 (0.00,10.35)
1.00 (0.32,3.11)	0.25 (0.00,41.01)	0.41 (0.04,4.10)	4.12 (0.04,437.21)	5.24 (0.04,614.11)	O	0.61 (0.05,8.12)	0.39 (0.02,6.92)	0.76 (0.06,8.96)
0.37 (0.11,1.19)	0.41 (0.00,45.70)	0.68 (0.20,2.26)	6.77 (0.10,467.70)	8.62 (0.11,663.40)	1.64 (0.12,21.96)	P	0.64 (0.08,5.28)	1.24 (0.27,5.63)
1.93 (0.02,194.87)	0.63 (0.00,83.74)	1.06 (0.19,5.98)	10.56 (0.13,872.53)	13.44 (0.15,1232.29)	2.56 (0.14,45.49)	1.56 (0.19,12.84)	Q	1.94 (0.27,13.75)
3.17 (0.05,207.45)	0.33 (0.00,34.41)	0.55 (0.22,1.36)	5.44 (0.08,349.50)	6.93 (0.10,496.66)	1.32 (0.11,15.64)	0.80 (0.18,3.64)	0.52 (0.07,3.65)	R
**Esophagitis**								
A	1.45 (0.02,84.99)	0.87 (0.01,53.97)	3.53 (0.05,269.73)	**0.27 (0.10,0.70)**	1.00 (0.11,8.74)	0.27 (0.06,1.18)	1.77 (0.02,153.66)	1.02 (0.01,73.30)
0.95 (0.47,1.90)	0.82 (0.01,54.13)	3.34 (0.04,269.81)	**0.25 (0.08,0.83)**	**0.26 (0.07,0.94)**	1.68 (0.02,153.46)	0.97 (0.01,73.38)	0.78 (0.00,123.06)	1.52 (0.02,111.40)
0.66 (0.03,13.01)	0.57 (0.00,86.52)	2.34 (0.01,417.84)	0.18 (0.01,4.04)	0.18 (0.01,4.96)	1.17 (0.01,233.17)	0.68 (0.00,114.76)	0.54 (0.00,173.80)	1.06 (0.01,175.17)
0.99 (0.53,1.85)	0.86 (0.01,51.12)	3.51 (0.05,256.05)	**0.26 (0.08,0.84)**	0.27 (0.05,1.33)	1.76 (0.02,146.05)	1.02 (0.01,69.53)	0.82 (0.01,118.37)	1.60 (0.02,105.47)
1.11 (0.21,5.75)	0.96 (0.01,81.94)	3.91 (0.04,403.92)	0.30 (0.04,1.99)	0.30 (0.03,2.73)	1.96 (0.02,228.22)	1.13 (0.01,110.23)	0.91 (0.00,178.52)	1.78 (0.02,167.66)
0.33 (0.01,13.15)	0.29 (0.04,1.88)	1.17 (0.12,11.58)	0.09 (0.00,3.97)	0.09 (0.00,4.73)	0.59 (0.05,7.34)	0.34 (0.04,2.96)	0.27 (0.01,8.23)	0.53 (0.07,4.35)
1.58 (0.16,15.63)	1.58 (0.02,117.79)	6.44 (0.07,583.98)	0.49 (0.10,2.33)	0.49 (0.09,2.57)	3.22 (0.03,331.08)	1.87 (0.02,159.09)	1.50 (0.01,262.31)	2.93 (0.04,241.74)
1.82 (0.53,6.26)	0.25 (0.01,7.44)	1.03 (0.03,38.80)	0.08 (0.01,1.02)	0.08 (0.00,1.30)	0.52 (0.01,22.64)	0.30 (0.01,10.41)	0.24 (0.00,19.91)	0.47 (0.01,15.68)
0.29 (0.03,3.15)	0.60 (0.29,1.21)	2.44 (0.55,10.81)	0.18 (0.00,12.10)	0.19 (0.00,14.19)	1.22 (0.20,7.62)	0.71 (0.19,2.57)	0.57 (0.03,10.62)	1.11 (0.34,3.58)
0.69 (0.01,40.50)	K	**4.08 (1.10,15.11)**	0.31 (0.00,21.49)	0.31 (0.00,25.16)	2.05 (0.38,11.05)	1.18 (0.40,3.48)	0.95 (0.06,16.29)	1.86 (0.73,4.71)
1.16 (0.02,72.08)	**0.24 (0.07,0.91)**	L	0.08 (0.00,6.41)	0.08 (0.00,7.46)	0.50 (0.06,4.23)	0.29 (0.05,1.58)	0.23 (0.01,5.32)	0.46 (0.09,2.27)
0.28 (0.00,21.61)	3.24 (0.05,226.29)	13.25 (0.16,1125.28)	M	1.02 (0.17,5.91)	6.64 (0.07,639.11)	3.84 (0.05,306.27)	3.08 (0.02,509.76)	6.03 (0.08,465.14)
**3.75 (1.43,9.87)**	3.20 (0.04,257.01)	13.05 (0.13,1270.24)	0.99 (0.17,5.73)	N	6.54 (0.06,718.82)	3.78 (0.04,346.38)	3.04 (0.02,565.54)	5.94 (0.07,526.60)
1.00 (0.11,8.74)	0.49 (0.09,2.64)	2.00 (0.24,16.88)	0.15 (0.00,14.51)	0.15 (0.00,16.82)	O	0.58 (0.08,4.28)	0.46 (0.02,12.65)	0.91 (0.13,6.23)
3.69 (0.85,16.09)	0.85 (0.29,2.48)	3.45 (0.63,18.81)	0.26 (0.00,20.79)	0.26 (0.00,24.23)	1.73 (0.23,12.79)	P	0.80 (0.04,16.78)	1.57 (0.38,6.52)
0.56 (0.01,49.05)	1.05 (0.06,18.05)	4.30 (0.19,98.20)	0.32 (0.00,53.64)	0.33 (0.00,61.34)	2.15 (0.08,58.65)	1.25 (0.06,26.02)	Q	1.96 (0.10,38.92)
0.98 (0.01,69.96)	0.54 (0.21,1.36)	2.20 (0.44,10.94)	0.17 (0.00,12.78)	0.17 (0.00,14.92)	1.10 (0.16,7.55)	0.64 (0.15,2.64)	0.51 (0.03,10.16)	R
**Nausea/vomiting**								
A	0.32 (0.01,8.43)	0.79 (0.02,27.70)	1.88 (0.84,4.21)	1.00 (0.18,5.63)	0.48 (0.03,7.15)			
2.97 (0.26,34.22)	2.33 (0.03,175.47)	5.59 (0.43,73.36)	1.41 (0.44,4.54)	9.78 (0.12,769.87)	0.62 (0.01,55.19)			
1.27 (0.69,2.36)	1.00 (0.03,33.41)	2.40 (0.87,6.61)	0.60 (0.04,9.76)	4.19 (0.12,148.08)	0.27 (0.01,10.90)			
0.54 (0.05,6.15)	0.42 (0.01,31.74)	1.02 (0.08,13.20)	0.26 (0.01,9.81)	1.78 (0.02,139.28)	0.11 (0.00,9.99)			
1.96 (0.10,37.90)	1.54 (0.21,11.10)	3.68 (0.17,79.49)	0.93 (0.02,51.62)	6.44 (0.81,51.28)	0.41 (0.04,4.16)			
3.51 (0.08,150.97)	0.20 (0.00,17.07)	0.49 (0.03,7.64)	**0.12 (0.03,0.56)**	0.86 (0.01,74.83)	0.05 (0.00,5.35)			
0.26 (0.02,3.59)	2.88 (0.20,40.87)	6.90 (0.56,84.94)	1.74 (0.05,64.09)	12.06 (0.79,184.35)	0.77 (0.04,14.16)			
3.66 (0.34,39.49)	1.88 (0.41,8.69)	4.51 (0.16,124.18)	1.14 (0.02,76.35)	**7.88 (1.51,41.20)**	0.50 (0.07,3.53)			
2.39 (0.10,59.69)	2.49 (0.62,9.90)	5.96 (0.20,175.25)	1.50 (0.02,106.30)	**10.43 (2.29,47.54)**	0.66 (0.11,4.16)			
3.16 (0.12,84.40)	L	2.40 (0.06,92.51)	0.60 (0.01,53.22)	4.19 (0.54,32.66)	0.27 (0.03,2.66)			
1.27 (0.04,44.88)	0.42 (0.01,16.08)	M	0.25 (0.01,4.27)	1.75 (0.04,71.14)	0.11 (0.00,5.21)			
0.53 (0.24,1.19)	1.65 (0.02,145.44)	3.96 (0.23,67.06)	N	6.93 (0.08,637.15)	0.44 (0.00,45.49)			
1.00 (0.18,5.63)	0.24 (0.03,1.86)	0.57 (0.01,23.25)	0.14 (0.00,13.25)	O	**0.06 (0.01,0.69)**			
2.10 (0.14,31.66)	3.76 (0.38,37.49)	9.01 (0.19,422.91)	2.27 (0.02,234.99)	**15.76 (1.45,170.99)**	R			

A, CCRT (cisplatin + etoposide); B, CCRT (carboplatin + paclitaxel); C, CCRT (pemetrexed + carboplatin); *CCRT* Concurrent chemoradiotherapy, *CI* Confidence interval; D, CCRT (pemetrexed + cisplatin); E, CCRT (docetaxel + cisplatin); F, CCRT (S-1 + cisplatin); G, CCRT (mitomycin + vindesine + cisplatin); H, CCRT (cisplatin + vinorelbine); I, CCRT (cisplatin); J, CCRT (etoposide + cisplatin + amifostine); K, RT; L, CCRT (5-FU); *LA-NSCLC* Locally advanced non-small cell lung cancer; M, CCRT (paclitaxel + cisplatin); N, CCRT (irinotecan + carboplatin); *NMA* Network meta-analysis; O, CCRT (nedaplatin); *OR* Odds radios; P, CCRT (carboplatin + etoposide); Q, CCRT (paclitaxel); R, CCRT (carboplatin)

### Network meta-analyses for non-hematological toxicities of different CCRT regimens

The results of network meta-analyses in non-hematological toxicities and the network evidence plots were shown in Table 10 and Fig. 2B. The results displayed that in terms of non-hematological toxicities, the incidences of pneumonia with CCRT (cisplatin + vinorelbine) and CCRT (irinotecan + carboplatin) were higher than CCRT (cisplatin + etoposide) (OR = 5.65, 95% CI = 1.82–17.56; OR = 2.14, 95% CI = 1.10–4.16; respectively). The incidences of pneumonia with CCRT (carboplatin + paclitaxel), CCRT (docetaxel + cisplatin), CCRT (mitomycin + vindesine + cisplatin), CCRT (paclitaxel + cisplatin), and CCRT (irinotecan + carboplatin) were higher than CCRT (etoposide + cisplatin + amifostine) (OR = 17.61, 95% CI = 1.62–191.58; OR = 46.79, 95% CI = 2.16–1013.71; OR = 44.08, 95% CI = 3.72–521.75; OR = 16.68, 95% CI = 1.68–165.50; OR = 21.23, 95% CI = 1.76–255.84; respectively). The incidence of pneumonia with CCRT (mitomycin + vindesine + cisplatin) was higher than CCRT (pemetrexed + cisplatin) (OR = 6.89, 95% CI = 1.42–33.51). The incidences of esophagitis with CCRT (cisplatin + etoposide) and CCRT (carboplatin + paclitaxel) were higher than CCRT (irinotecan + carboplatin) (OR = 3.75, 95% CI = 1.43–9.87; OR = 3.90, 95% CI = 1.07–14.28; respectively). The incidences of esophagitis with CCRT (carboplatin + paclitaxel) and CCRT (pemetrexed + cisplatin) were higher than CCRT (paclitaxel + cisplatin) (OR = 3.96, 95% CI = 1.20–13.03; OR = 3.78, 95% CI = 1.20–11.94; respectively). The incidence of esophagitis with CCRT (5-FU) was higher than RT (OR = 4.08, 95% CI = 1.10–15.11). The incidence of nausea/vomiting with CCRT (mitomycin + vindesine + cisplatin) was higher than CCRT (carboplatin + paclitaxel) and CCRT (irinotecan + carboplatin) (OR = 11.41, 95% CI = 4.38–29.77; OR = 8.10, 95% CI = 1.78–36.76; respectively). The incidence of the nausea/vomiting with CCRT (nedaplatin) was higher than CCRT (cisplatin), RT, and CCRT (carboplatin) (OR = 7.88, 95% CI = 1.51–41.20; OR = 10.43, 95% CI = 2.29–47.54; OR = 15.76, 95% CI = 1.45–170.99; respectively).

### Cumulative probability of efficacies and toxicities of different CCRT regimens

**Fig. 3:**
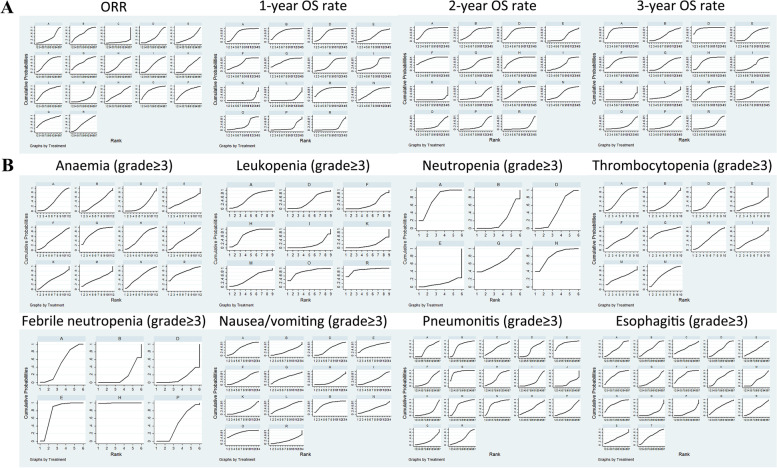
The SUCRA plots of efficacies and toxicities of different CCRT regimens. **A** The SUCRA plots of efficacies of different CCRT regimens. **B** The SUCRA plots of toxicities of different CCRT regimens. *SUCRA curve was used to compare the SUCRA value of different CCRT regimens to ascertain the efficacies or the toxicities ranks, the larger the SUCRA value, the better the efficacy or the lower the toxicity. A* = *CCRT (cisplatin* + *etoposide). B* = *CCRT (carboplatin* + *paclitaxel). C* = *CCRT (pemetrexed* + *carboplatin). CCRT* = *concurrent chemoradiotherapy. D* = *CCRT (pemetrexed* + *cisplatin). E* = *CCRT (docetaxel* + *cisplatin). F* = *CCRT (S-1* + *cisplatin). G* = *CCRT (mitomycin* + *vindesine* + *cisplatin). H* = *CCRT (cisplatin* + *vinorelbine). I* = *CCRT (cisplatin). K* = *RT. L* = *CCRT (5-FU). M* = *CCRT (paclitaxel* + *cisplatin). N* = *CCRT (irinotecan* + *carboplatin). O* = *CCRT (nedaplatin). ORR* = *overall response rate. OS* = *overall survival. P* = *CCRT (carboplatin* + *etoposide). Q* = *CCRT (paclitaxel). R* = *CCRT (carboplatin). SUCRA* = *surface under the cumulative ranking*

As shown in Table 11 and Fig. 3, the SUCRA values of different CCRT regimens demonstrated that in terms of efficacies, the ORR of CCRT (nedaplatin) ranked the highest (75.9%), followed by CCRT (paclitaxel) (73.2%). The 1-year OS rate of CCRT (paclitaxel + cisplatin) ranked the highest (85.9%), followed by CCRT (paclitaxel + carboplatin) (78.4%), CCRT (mitomycin + vindesine + cisplatin) (76.5%), CCRT (etoposide + cisplatin) (72.5%), and CCRT (pemetrexed + cisplatin) (70.4%). The 2-year OS rate of CCRT (S-1 + cisplatin) ranked the highest (83.8%), followed by CCRT (etoposide + cisplatin) (79.7%), CCRT (pemetrexed + cisplatin) (79.7%), and CCRT (carboplatin + paclitaxel) (68.6%). The 3-year OS rate of CCRT (pemetrexed + cisplatin) ranked the highest (95.1%), followed by CCRT (etoposide + cisplatin) (89.4%). As for toxicities, the incidences of anemia and febrile neutropenia with CCRT (pemetrexed + cisplatin) ranked the lowest (26.7%, 12.0%, respectively), followed by CCRT (paclitaxel + carboplatin) (39.3%, 16.7%, respectively). The incidences of neutropenia and thrombocytopenia with CCRT (docetaxel + cisplatin) ranked the lowest (10.3%, 27.4%, respectively), followed by CCRT (paclitaxel + carboplatin) (21.6%, 32.9%, respectively). The incidences of leukopenia with CCRT (cisplatin) and RT ranked the lowest (17.9%, 17.9%, respectively). The incidence of nausea/vomiting with CCRT (carboplatin) ranked the lowest (23.2%), followed by CCRT (cisplatin + vinorelbine) (26.1%). The incidence of esophagitis with CCRT (paclitaxel + cisplatin) ranked the lowest (16.9%), and the incidence of pneumonitis with CCRT (etoposide + cisplatin + amifostine) ranked the lowest (17.7%).

**Table 11:** Cumulative probability of efficacies and toxicities of different CCRT regimens in the treatment of LA-NSCLC

Treatment	SUCRA values (%)											
	ORR	1-year OS rate	2-year OS rate	3-year OS rate	Anemia	Leukopenia	Neutropenia	Thrombocytopenia	Febrile neutropenia	Nausea/vomiting	Esophagitis	Pneumonitis
A	32.0	72.5	79.7	89.4	44.3	55.2	76.3	58.2	49.4	59.1	51.0	49.0
B	64.1	78.4	68.6	49.1	39.3	NR	21.6	32.9	16.7	32.4	53.7	69.2
C	7.3	NR	NR	NR	NR	NR	NR	NR	NR	NR	56.2	56.3
D	37.2	70.4	79.7	**95.1**	**26.7**	40.7	45.0	44.2	**12.0**	50.1	50.1	45.6
E	33.2	45.3	33.0	51.4	42.5	NR	**10.3**	**27.5**	77.9	73.1	47.1	86.8
F	64.6	62.7	**83.8**	77.8	47.7	25.5	NR	47.9	NR	45.4	73.5	36.7
G	67.3	76.5	51.1	47.1	77.9	NR	65.8	81.3	NR	59.1	33.1	90.1
H	47.8	52.4	81.8	75.4	53.7	71.4	NR	48.2	99.8	26.1	75.2	54.8
I	48.8	49.7	48.7	41.8	48.3	**17.9**	NR	43.5	NR	40.1	55.2	26.9
J	NR	NR	NR	NR	NR	NR	NR	NR	NR	NR	NR	**17.7**
K	38.3	7.9	3.5	5.2	42.6	**17.9**	NR	NR	NR	29.8	36.9	23.0
L	70.8	11.2	27.9	28.4	NR	NR	NR	NR	NR	56.4	76.9	NR
M	19.8	**85.9**	68.2	66.3	46.6	48.4	NR	51.0	NR	77.2	**16.9**	69.8
N	53.3	62.3	56.2	43.5	55.6	NR	81.0	65.3	44.2	43.6	19.5	74.1
O	**75.9**	38.4	27.3	22.9	NR	82.8	NR	NR	NR	84.6	59.4	45.6
P	68.2	19.1	25.7	29.5	NR	NR	NR	NR	NR	NR	44.1	35.5
Q	73.2	NR	NR	NR	74.7	NR	NR	NR	NR	NR	43.2	26.8
R	48.1	17.3	14.8	27.3	NR	90.2	NR	NR	NR	**23.2**	58.2	42.4

A, CCRT (cisplatin + etoposide); B, CCRT (carboplatin + paclitaxel); C, CCRT (pemetrexed + carboplatin); *CCRT* Concurrent chemoradiotherapy; D, CCRT (pemetrexed + cisplatin); E, CCRT (docetaxel + cisplatin); F, CCRT (S-1 + cisplatin); G, CCRT (mitomycin + vindesine + cisplatin); H, CCRT (cisplatin + vinorelbine); I, CCRT (cisplatin); J, CCRT(etoposide + cisplatin + amifostine); K, RT; L, CCRT (5-FU); *LA-NSCLC* Locally advanced non-small cell lung cancer; M, CCRT (paclitaxel + cisplatin); N, CCRT (irinotecan + carboplatin); *NR* Not report; O, CCRT (nedaplatin); *ORR* Objective response rate, *OS* Overall survival; P, CCRT (carboplatin + etoposide); Q, CCRT (paclitaxel); R, CCRT (carboplatin); *SUCRA* Surface under the cumulative ranking

### Cluster analyses regarding efficacies and toxicities in the included studies

**Fig. 4:**
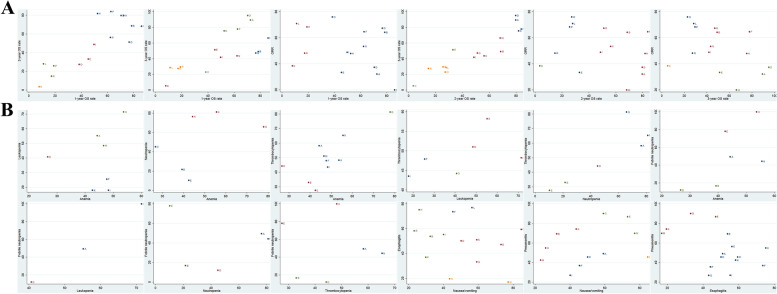
Clustered ranking plots based on SUCRA values of efficacies and efficacies with different CCRT regimens in the treatment of LA-NSCLC. **A** Clustered ranking plots based on SUCRA values of efficacies with different CCRT regimens in the treatment of LA-NSCLC. **B** Clustered ranking plots based on SUCRA values of toxicities with different CCRT regimens in the treatment of LA-NSCLC. *Studies on the upper right corner have better efficacy or higher toxicity. A* = *CCRT (cisplatin* + *etoposide). B* = *CCRT (carboplatin* + *paclitaxel). C* = *CCRT (pemetrexed* + *carboplatin). CCRT* = *Concurrent Chemoradiotherapy. D* = *CCRT (pemetrexed* + *cisplatin). E* = *CCRT (docetaxel* + *cisplatin). F* = *CCRT (S-1* + *cisplatin). G* = *CCRT (mitomycin* + *vindesine* + *cisplatin). H* = *CCRT (cisplatin* + *vinorelbine). I* = *CCRT (cisplatin). K* = *RT. L* = *CCRT (5-FU). LA-NSCLC* = *locally advanced non-small cell lung cancer. M* = *CCRT (paclitaxel* + *cisplatin). N* = *CCRT (irinotecan* + *carboplatin). O* = *CCRT (nedaplatin). ORR* = *overall response rate. OS* = *overall survival. P* = *CCRT (carboplatin* + *etoposide). Q* = *CCRT (paclitaxel). R* = *CCRT (carboplatin). SUCRA* = *surface under the cumulative ranking*

The cluster analyses based on SUCRA values indicated that the regimens with CCRT (cisplatin + etoposide), CCRT (carboplatin + paclitaxel), CCRT (pemetrexed + cisplatin), CCRT (S-1 + cisplatin), and CCRT (cisplatin + vinorelbine) had relatively better efficacies compared with other regimens (Fig. 4A). As for toxicities of different CCRT regimens, the hematological toxicities of CCRT (carboplatin + paclitaxel), CCRT (pemetrexed + cisplatin), and CCRT (docetaxel + cisplatin) were relatively lower (Fig. 4B). However, the differences of non-hematological toxicities with different CCRT regimens was not significant (Fig. 4B).

### Publication bias regarding efficacy and toxicity in the included studies

**Fig. 5:**
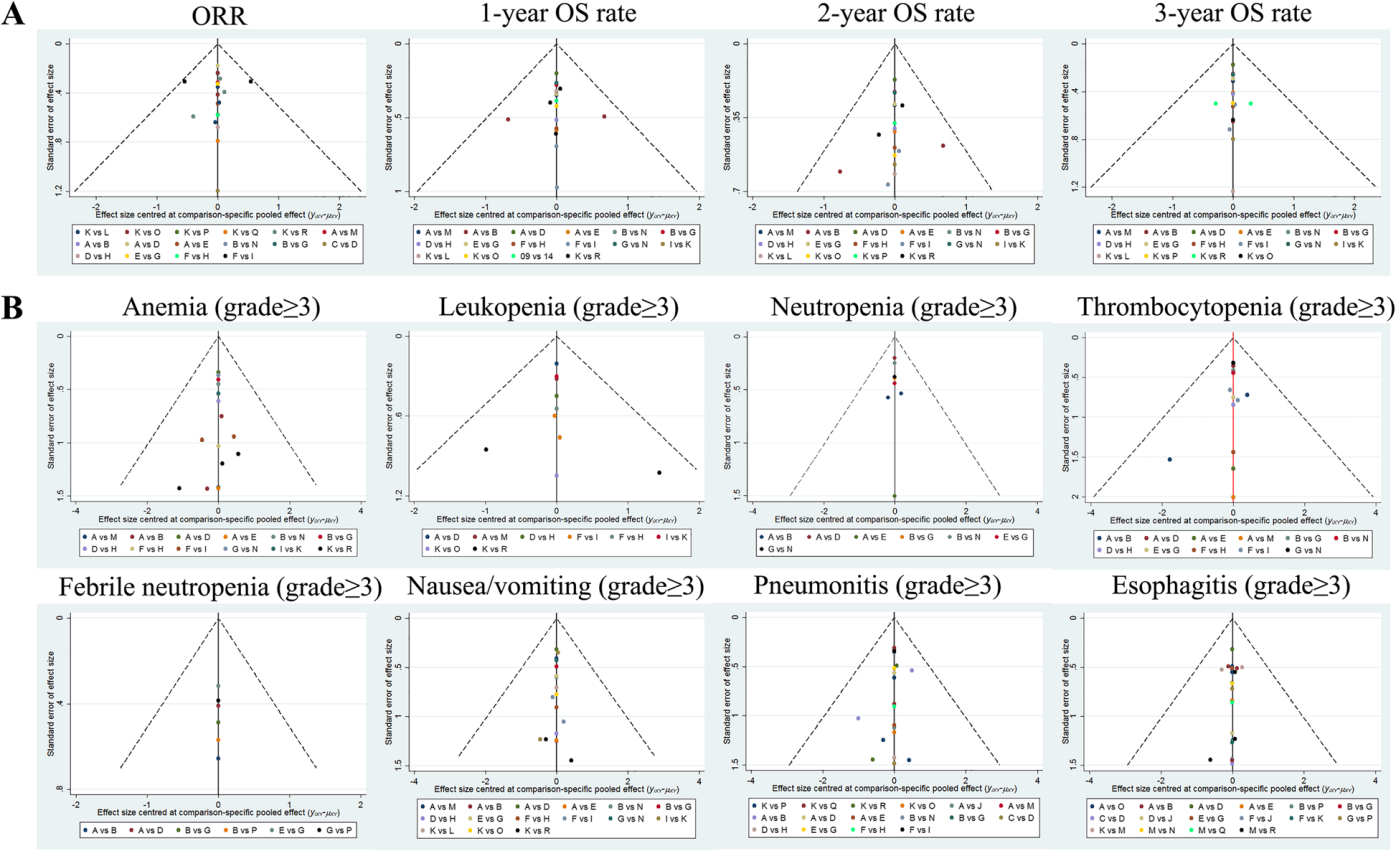
Comparison-adjusted funnel plots of efficacies and toxicities of different CCRT regimens. **A** Comparison-adjusted funnel plots of efficacies of different CCRT regimens. **B** Comparison-adjusted funnel plots of toxicities of different CCRT regimens. *A* = *CCRT (cisplatin* + *etoposide). B* = *CCRT (carboplatin* + *paclitaxel). C* = *CCRT (pemetrexed* + *carboplatin). CCRT* = *concurrent chemoradiotherapy. D* = *CCRT (pemetrexed* + *cisplatin). E* = *CCRT (docetaxel* + *cisplatin). F* = *CCRT (S-1* + *cisplatin). G* = *CCRT (mitomycin* + *vindesine* + *cisplatin). H* = *CCRT (cisplatin* + *vinorelbine). I* = *CCRT (cisplatin). K* = *RT. L* = *CCRT (5-FU). M* = *CCRT (paclitaxel* + *cisplatin). N* = *CCRT (irinotecan* + *carboplatin). O* = *CCRT (nedaplatin). ORR* = *overall response rate. OS* = *overall survival. P* = *CCRT (carboplatin* + *etoposide). Q* = *CCRT (paclitaxel). R* = *CCRT (carboplatin)*

The comparison-adjusted funnel plots of efficacy and toxicity of CCRT regimens showed that there were no publication bias among the included studies (Fig. 5).

## Discussion

LA-NSCLC keeps a high incidence and mortality around the world. Although many kinds of CCRT regimens have been utilized for treating it, the OS is still poor because of the high probability of lurks and recurrence. To give valuable suggestions for treatments through comparing the efficacy and safety, we conducted the NMAs among 18 CCRT regimens with 14 drugs commonly used for LA-NSCLC, including cisplatin, docetaxel, pemetrexed, paclitaxel, carboplatin, etoposide, and others. The main advantage of our study over published systematic reviews is that we could compare a variety of CCRT regimens simultaneously by applying the network method.

The results of efficacy conducted in the present study revealed that ORR of CCRT (nedaplatin) were higher than other regimens. Nedaplatin is a second generation platinum analog, and its mechanism of antitumor action is suggested as its interference on DNA adducts formation by affecting DNA damage repairing proteins, transcription factors and DNA polymerases [42]. It has been verified that nedaplatin concurrent radiotherapy for treating stage III/IV non-surgical patients with NSCLC showed a good curative effect of better ORR and well-tolerated [20], which is similar with the results of our NMA. However, it had a bad performance in known adverse events including leukopenia and nausea/vomiting, which suggested that the CCRT (nedaplatin) regimen may have a good efficacy and a high incidence of toxicity for the treatment in LA-NSCLC.

Focusing on the long-term efficacy, 1-year OS rate, 2-year OS rate, and 3-year OS rate of CCRT (etoposide + cisplatin), CCRT (paclitaxel + carboplatin), and CCRT (pemetrexed + cisplatin) were relatively higher. Moreover, the toxicities of CCRT (paclitaxel + carboplatin) and CCRT (pemetrexed + cisplatin) were relatively lower. Cisplatin, as an assistant drug for chemotherapy, was often combined with other drugs to treat cancer but was also combined by some side effects [43]. Pemetrexed is a potent inhibitor of thymidylate synthase [44] and other folate-dependent enzymes, including dihydrofolate reductase and glycinamide ribonucleotide formyl transferase [45]. Pemetrexed was formerly approved as a single agent for second-line treatment of advanced NSCLC [46]. Some studies have suggested that the combination of cisplatin and pemetrexed has promising activity and tolerability in locally advanced unresectable Stage III NSCLC when combined with RT [47–49]. Mornex F et al. [47] evaluated chemotherapy with cisplatin and pemetrexed and found that it is well tolerated and appears to be the only third-generation agent that can likely be recommended safely at full dose with concurrent RT, avoiding compromise on activity against distant disease while optimizing local control. The pemetrexed + cisplatin regimen are known to cause severe side effects, but our study indicated that the incidences of anemia and febrile neutropenia with CCRT (pemetrexed + cisplatin) were lower than other regimens, and other toxicities had not significant differences with other regimens. The doublet combinations of platinum compounds (cisplatin or carboplatin) with taxanes (paclitaxel or docetaxel) are also the reference regimens for NSCLC [50]. The carboplatin plus paclitaxel regimen is one of the most commonly used regimens, and there is considerable interest in the combination of carboplatin + paclitaxel and RT for the treatment of LA-NSCLC. Several studies had certified that CCRT (carboplatin + paclitaxel) had a better efficacy in the treatment of unresectable NSCLC [11, 51].

Subsequently, the cluster analysis of efficacies and toxicities of CCRT regimens demonstrated that CCRT (carboplatin + paclitaxel) and CCRT (pemetrexed + cisplatin) regimens had better efficacies and lower toxicities, may be the best regimens in treating LA-NSCLC. In addition, CCRT (cisplatin + etoposide), CCRT (S-1 + cisplatin), and CCRT (cisplatin + vinorelbine) also have relatively better efficacies, but the incidences of neutropenia with CCRT (cisplatin + etoposide), esophagitis with CCRT (S-1 + cisplatin), and leukopenia, febrile neutropenia, and esophagitis with CCRT (cisplatin + vinorelbine) were higher. Yamaguchi M et al. [52] reported that 74.2% of NSCLC patients treated with cisplatin plus etoposide regimen had incidence of neutropenia, which is similar to our study.

Despite the existence of dilemma in treatment of unresectable LA-NSCLC, the years since about 2010 have brought great progress in the understanding of the molecular mechanisms related to tumour immunology [53]. The discovery of these mechanisms has led to the development of several new drugs, including immune checkpoint inhibitors (ICIS) that specifically target PD-1, PD-L1, and CTLA-4 receptor, as well as drugs that target other regions of the immune system pathway. The use of ICIS as consolidation therapy within a curative-intent management plan for LA-NSCLC represents a promising strategy to improve the prognosis after CCRT. In NSCLC, the antitumour immunogenic effects of radiation might act as an adjuvant to checkpoint blockade. Theoretically, the combination of RT and ICIS could lead to enhanced responses by increasing the exposure or altering the presentation of tumour-related antigens to immune system cells. In a study of patients with advanced NSCLC treated with pembrolizumab, PFS and OS were longer in those who had previously received RT than in those who had not [54]. Moreover, compared with patients who received pembrolizumab alone, patients who were randomized to receive stereotactic body radiation therapy for a single metastasis before receiving pembrolizumab for advanced NSCLC experienced an improved response rate (41% vs. 19%) and PFS (6.4 months vs. 1.8 months; HR: 0.55; *P* = 0.04) [55]. The NICOLAS study is the first completed single-arm phase II trial in stage III NSCLC evaluating hierarchically first the safety and then the efficacy of adding nivolumab concurrently to standard definitive concurrent chemoradiotherapy, and it suggested that 44% of the cases being attributed to treatment toxicity [56]. Although there are many studies on the treatment measures after CCRT, the systematic analysis needs further research.

A few limitations should be mentioned. Firstly, due to the lack of available appropriate head-to-head RCTs, the quantity of studies included for one specific comparison was small, we could not compare all treatment interventions simultaneously for each complication in one network. Secondly, the sample size of some included RCTs were relatively small, which may affect the results. Thirdly, all of the data was extracted from published RCT studies, and individual patient data were not used. Therefore, the quality control of the data was difficult. Fourthly, the qualities of the included RCTs varied, however, our comparison-adjusted funnel plot did not exhibit any asymmetry, indicating that there were no significant publication bias. Finally, studies were conducted over a wide time period. With improvements in equipment and a greater understanding of quality metrics to augment the rate of detection of LA-NSCLC, it is possible that detection rates could have varied over time.

## Conclusion

In conclusion, for the treatment of LA-NSCLC, CCRT (pemetrexed + cisplatin) and CCRT (paclitaxel + carboplatin) resulted in better efficacies and lower toxicities simultaneously. The 3-year OS rate of CCRT (pemetrexed + cisplatin) ranked the highest. These findings may help clinicians in their choice of proper CCRT regimens for LA-NSCLC patients.

## Data Availability

All data generated or analyzed during this study are included in this published article.

## Abbreviations

CCRT: Concurrent chemoradiotherapy
CENTRAL: Cochrane central register of controlled trials
CI: Confidence interval
LA-NSCLC: Locally advanced non-small cell lung cancer
MTC: Multiple-treatments comparison
NMA: Network meta-analysis
OS: Newcastle–Ottawa Quality Assessment Scale
OR: Odds radio
ORR: Overall response rate
OS: Overall survival
RCT: Randomized controlled trials
RT: Radiation Therapy
RTOG: Radiation Therapy Oncology Group
SUCRA: Surface under the cumulative ranking
